# A concise overview of non-invasive intra-abdominal pressure measurement techniques: from bench to bedside

**DOI:** 10.1007/s10877-020-00561-4

**Published:** 2020-07-22

**Authors:** Salar Tayebi, Adrian Gutierrez, Ikram Mohout, Evelien Smets, Robert Wise, Johan Stiens, Manu L. N. G. Malbrain

**Affiliations:** 1 Department of Electronics and Informatics, Vrije Universiteit Brussel, Pleinlaan 2, 1050 Brussels, Belgium; 2 Adult Intensive Care, John Radcliffe Hospital, Oxford University Hospitals Trust, Oxford, England; 3 Discipline of Anaesthesia and Critical Care, School of Clinical Medicine, University of KwaZulu-Natal, Durban, South Africa; 4 Intensive Care Unit, ICU Director, University Hospital Brussel (UZB), Laarbeeklaan 101, 1090 Jette, Belgium; 5 Faculty of Medicine and Pharmacy, Vrije Universiteit Brussel (VUB), Laarbeeklaan 103, 1090 Jette, Belgium

**Keywords:** Non-invasive measurement, Intra-abdominal pressure, Abdominal compartment syndrome, Intensive care unit, Ultrasound, Microwave reflectometry

## Abstract

This review presents an overview of previously reported non-invasive intra-abdominal pressure (IAP) measurement techniques. Each section covers the basic physical principles and methodology of the various measurement techniques, the experimental results, and the advantages and disadvantages of each method. The most promising non-invasive methods for IAP measurement are microwave reflectometry and ultrasound assessment, in combination with an applied external force.

## Introduction

Around 25% of critically ill patients suffer from intra-abdominal hypertension (IAH), defined as a sustained increase in intra-abdominal pressure (IAP) equal to or above 12 mmHg. More than half of the patients hospitalized in intensive care units (ICUs) will develop IAH within the first week [1]. The presence of IAH significantly affects perfusion to abdominal organs [2] and can result in diminished organ perfusion, organ dysfunction, and depending on the degree of IAP, potentially multiple organ failure and death [3–5]. Furthermore, late detection of IAH can result in abdominal compartment syndrome (ACS), a fatal condition characterized by a sustained increase in IAP above 20 mmHg with new onset organ failure. Although the incidence of ACS has decreased over the last decades and is currently estimated around 3–5% in general ICU patients, the risk of ACS development should not be underestimated [2]. Monitoring the IAP is thus absolutely necessary for patients hospitalized in the ICU, as measuring IAP is knowing and understanding ACS.

Various direct and indirect techniques for IAP measurement have been suggested. Presently, the gold standard for IAP measurement advocated by the Abdominal Compartment Society (WSACS, www.wsacs.org, formerly known as the World Society of the Abdominal Compartment Syndrome) is an indirect measurement via the bladder, measured in the supine position after instilling a maximum of 25 ml into the bladder, with the zero reference level where the mid-axillary line crosses the iliac crest. This measurement technique is cumbersome, non-continuous, and carries a potential risk for infection. Therefore, other less-invasive indirect IAP measurement techniques have been suggested, with a primary focus on accuracy and continuity [6, 7]. All potential non-invasive IAP measurement techniques are reviewed in this paper. The first section examines the possibility of using a strain gauge, respiratory inductance plethysmography, or abdominal tensiometer. The second section relates to the application of ultrasound-based techniques for IAP monitoring. The third section discusses bio-electrical impedance and microwave reflectometry, while digital image correlation and the use of a wireless motility capsule are examined in the final section. Accordingly, this review paper can be used as a guideline for future studies; to promote research comparing the different existing techniques, and to encourage researchers to further refine the most promising techniques for non-invasive IAP measurement.

## Methods

We included all clinical and medical aspects in relation to the way how the intra-abdominal volume correlates with IAP within the abdominal cavity, with a focus on IAP and signal monitoring. A broad search of the English literature in PubMed and Medline using the terms of “intra-abdominal pressure” or “abdominal pressure” and “non-invasive” was performed. After analysis, the papers identified as relevant were included in this narrative review.

## Results

### Strain gauge, respiratory inductance plethysmography, and tensiometer

#### Strain gauge

A strain gauge is a device in which the electrical resistance varies in proportion to the amount of force applied. It converts force, pressure, and tension into a change in electrical resistance that can then be measured [8]. The working principle is based on an electrical resistance that changes as a function of deformation and is demonstrated practically by a Wheatstone Bridge. The gauge factor (GF) is the fundamental parameter that can be defined as a strain gauge’s sensitivity to strain [8]. The GF is equal to the ratio of fractional change in electrical resistance to the fractional change in length (strain):

1 $$GF = \frac{\Delta R/R}{{\Delta L/L}} = \frac{\Delta R/R}{\varepsilon }$$

In Eq. (1), *R* and *L* are the electrical resistance and original length, respectively.

$\varepsilon$ represents the strain which is defined as $\Delta L/L$.

**Fig. 1:**
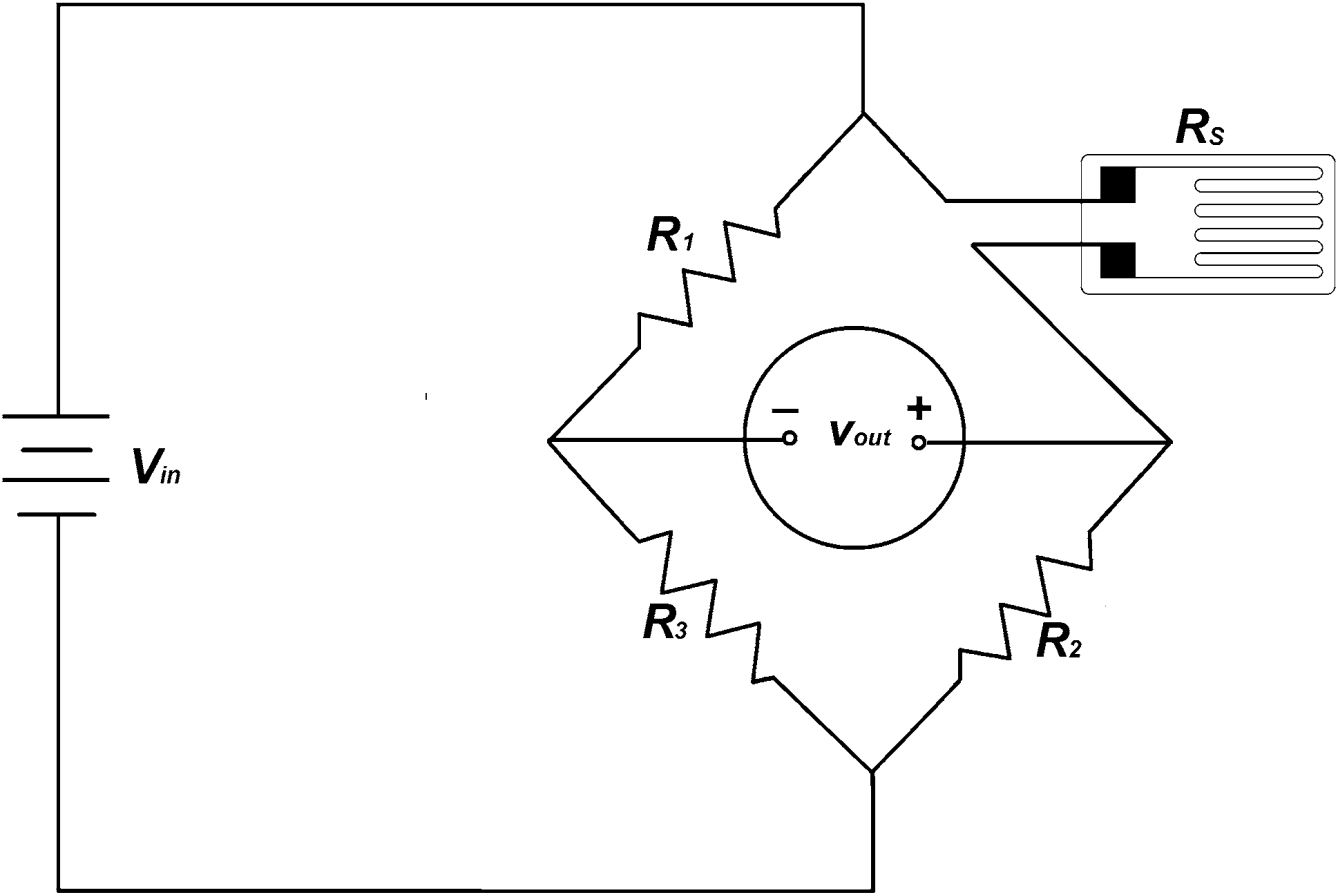
Wheatstone bridge strain gauge. In this circuit, *V*_in_ is the input voltage, *R*_1_, *R*_2_, *R*_3_, and R_S_ are resistors and *V*_out_ is the output voltage that fluctuates according to the changes in *R*_S_

In Fig. 1, *R*_S_ is the resistor and its value is a function of strain ($\Delta L/L)$, multiplied by GF (see Eq. 1). On the other hand, *V*_out_ represents a voltage value that fluctuates according to the changes in *R*_S_ (see Eq. 2)_._ Finally, the data is displayed through the voltage value that represents the change of deformation over time. Nowadays, strain gauges have been implemented in numerous medical devices. They are used as a tool to assist monitoring the movement and force applied to patients.

2 $$V_{out} = \frac{{R_{S} R_{3} - R_{1} R_{2} }}{{\left( {R_{S} + R_{2} } \right)\left( {R_{3} + R_{1} } \right)}}V_{in}$$

Boudewyns et al. studied respiratory effort by implementing strain gauges [9]. Strain gauges were used to detect rib cage and abdominal movement. Thoracic and abdominal strain gauges were positioned just below the axilla and at the umbilicus. The objective was to characterize apneas and examine the impact of sleep posture.

Boudewyns et al. also showed that strain gauges are sufficiently reliable when describing apneas in most patient populations [9]. However, movement artifacts affect the results, especially in obese patients. In approximately 10% of patients, the signal from the abdominal movement and ribcage was very poor [9], revealing early pros and cons for this technique.

Hodges et al. analyzed the effect of IAP on the human spine [10], hypothesizing that an increase in IAP would cause an extensor movement around L_3_ (third lumbar vertebrae). IAP was increased in the test subjects by means of tetanic stimulation of the phrenic nerve.

**Fig. 2:**
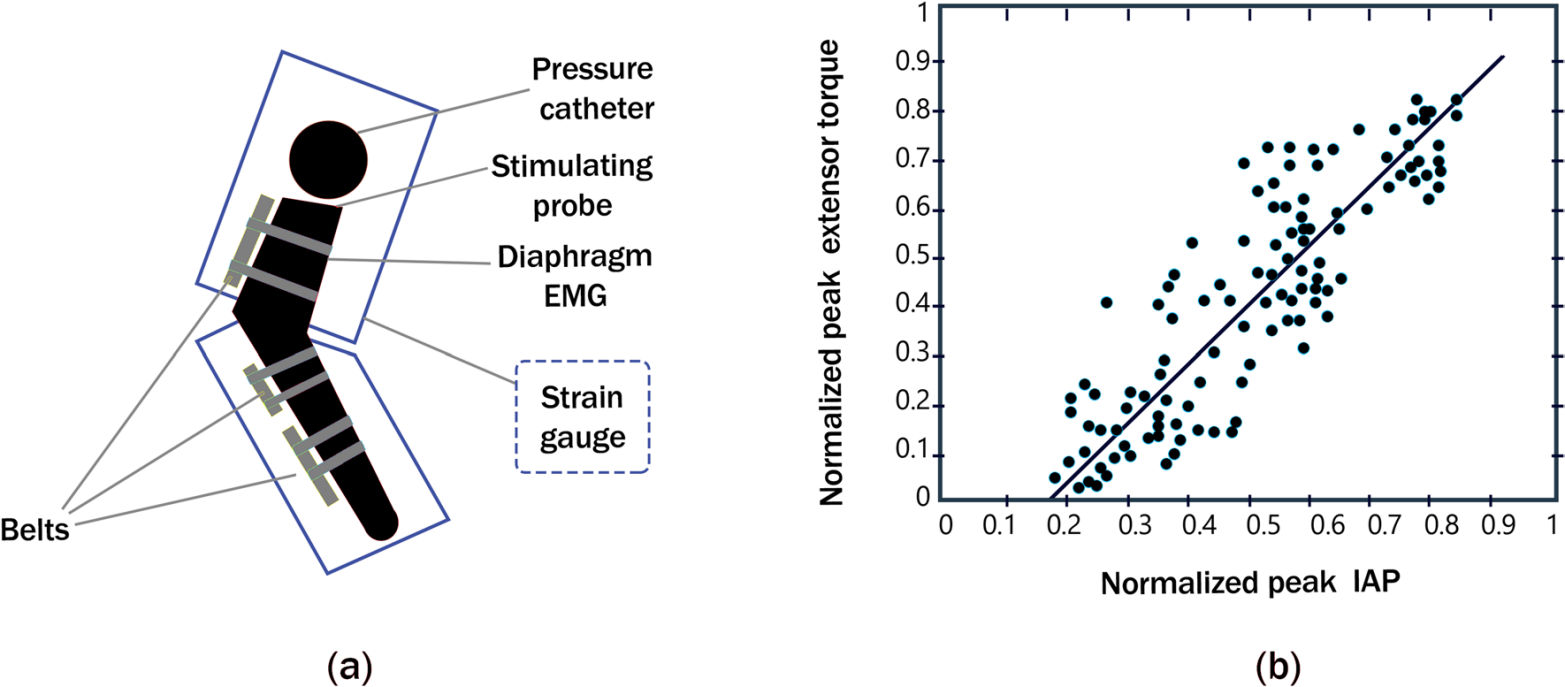
Summary of results of the study about the effect of IAP on the human spine (adapted with permission from Hodges et al. [10]). **a** Experimental set-up used in the study. By stimulating phrenic nerve, IAP is increased and the extensor torque around mid-lumbar level is measured by means of a strain gauge. Peak IAP values are achieved by using a pressure catheter which is known as the intragastric method **b** Normalized peak extensor torque as a function of peak IAP (R = 0.86, P < 0.01) (adapted from Hodges et al. [10])

Trunk extension was calculated by measuring the extension force with a strain gauge (see Fig. 2a [10]). Also, the correlation between IAP and trunk extension was investigated by measuring the IAP invasively.

Results showed a likely correlation between the amplitude of the IAP (increase) and the amplitude of the extensor torque at mid-lumbar level (correlation coefficient (R) of 0.86 and p < 0.01) (see Fig. 2b [10]). This study demonstrated that this strain gauge technique was capable of providing valuable information (regarding IAP) despite artifacts.

Strain gauges are simple and low-cost devices. They are capable of providing a great deal of interesting information regarding patient position and further movement (e.g. a gyroscope). Negative aspects include motion artifacts and other forms of distortion that can affect results. Strain gauges should not be used as standalone technology for measuring IAP as accuracy remains an issue. Rather, they should be considered in combination with other technology, to enhance accuracy.

#### Respiratory inductance plethysmography (RIP)

**Fig. 3:**
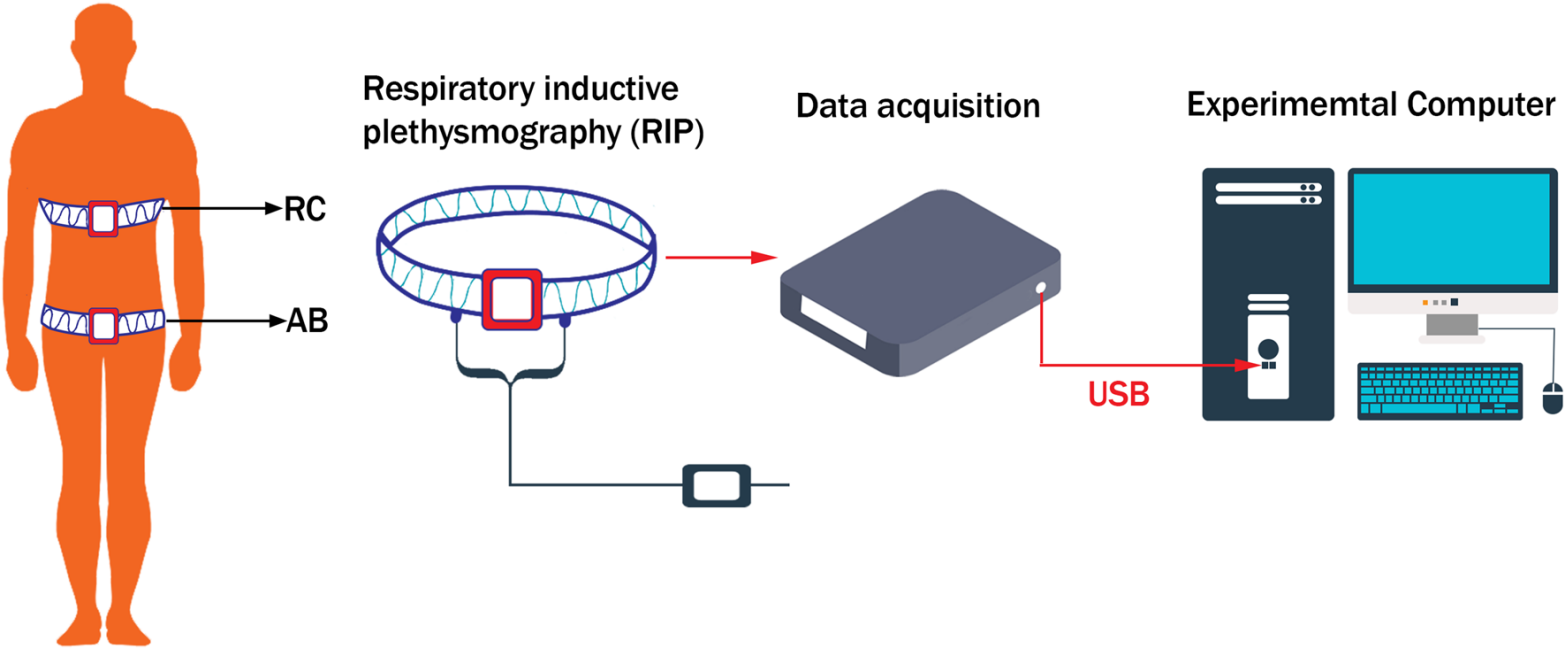
The general principle of respiratory inductance plethysmography (RIP). Firstly, the coils should be positioned around the rib cage (RC) and abdomen (AB). Finally, by data acquisition, the changes in the length of AB and RC coils will be achieved (adapted with permission from Chen et al. [11])

Other techniques to study interactions between the abdomen and thorax are combined thoracic and abdominal plethysmography and electrical impedance tomography [12]. Respiratory inductance plethysmography (RIP) was first introduced as a non-invasive respiratory assessment system in 1977 by Cohn. It consisted of two winding wire coils within elastic bands and encircling the rib cage (RC) and abdomen (AB) [13]. The inductance of the coils is a function of the cross-sectional area of AB and RC [14]. A general schematic representation for a RIP setup is shown in Fig. 3 [11]. Working with RIP allows the simultaneous recordings of pressure and volume excursions within the abdomen and thorax. These can be used to identify IAP and movements that can be caused by alterations in compliance of these compartments. The chest wall motions can be converted to volume changes. The relationship between RC and AB signals and tidal volume (TV), can be described by the following equation:

3 $$TV \, = \alpha \times \, \Delta L\left( {RC} \right) \, + \beta \times\, \Delta L\left( {AB} \right)$$

Here, *α* and *β* are the coefficients describing the relationship between motion and volume changes in the rib cage and the abdominal compartment, and *ΔL*(RC) and *ΔL*(AB) are the dimensional changes of the rib cage and abdomen. The IAV can be calculated as follows:

4 $$IAV \, = k \times \, [(\alpha/\beta) \, \times \, \Delta L\left( {RC} \right) \, + \, \Delta L\left( {AB} \right)]$$

**Fig. 4:**
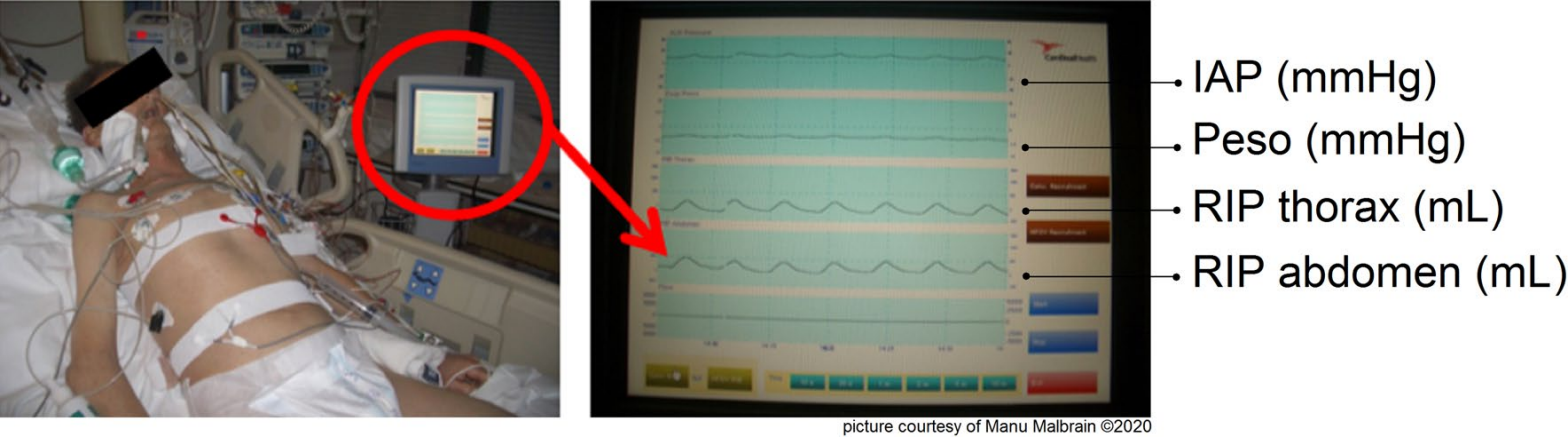
The tracings achieved by respiratory inductance plethysmography (RIP). The first signal shows IAP in mmHg versus time in seconds. Peso is the esophageal pressure in mmHg that is shown as the second signal. The last two signals are related to thorax and abdominal volume changes that have been assessed by thorax and abdominal respiratory inductance plethysmography (RIP). Sample tracings obtained with BiCore monitor (Cardinal Health, Dublin, Ohio, United States)

α/β is the weighting coefficient and κ is a factor converting a change in dimension to volume in litres. In such a way, one can determine IAP by dividing IAV by abdominal compliance (*C*_ab_; defined as the change in IAV per change in IAP and expressed in ml/mmHg) [15]. Generally, measurement of *C*_ab_ is difficult at the bedside and can only be done in case of change in IAV. Finally, by plotting IAV versus IAP, the effects of the different actions of the thoracic and abdominal compartment can be studied (see Fig. 4).

In a study involving 5 normal subjects, *C*_ab_ was measured using RIP. In the supine position, *C*_ab_ was 250 ± 100 ml(mmHg)^−1^. Changing to an upright position reduced *C*_ab_ to 48 ± 20 ml(mmHg)^−1^ [15].

In another study of three healthy subjects, fluid was instilled into the stomach and subsequently withdrawn. Volume changes of abdomen, lung, and rib cage were assessed using magnetometry. In the 70° head-of-bed (HOB) position the mean *C*_ab_ was 49 ± 20 ml (mmHg)^−1^. Interestingly, the gastric distension caused changes in the volume of the various compartments; 33% of the volume changes were linked to a decrease in lung volume, 40% to an increase in rib cage volume, and 26% to an increase in abdominal volume. The authors concluded that the interactions between the rib cage, abdomen, and diaphragm aim to limit large changes in end-expiratory lung volume, even in the face of abdominal distension [16].

Several techniques such as electric impedance plethysmography (EIP), RIP, magnetometers/strain gauge sensors, and piezoresistive materials can be used as wearable respiratory monitoring to diagnose a variety of diseases. However, RIP has the advantage of better accuracy, sensitivity and safety, compared with the other techniques [17–19]. Despite these advantages, the simplicity and low-cost, it seems logical to combine RIP with another monitoring system for better results.

#### Tensiometry

Tensiometry is another non-invasive technique for estimation of IAP. The degree of indentation at the site where force is applied during palpation of the abdomen can be measured. Abdominal palpation examines intra-abdominal, passive and active muscle tension. Identifying increased muscle tension may be useful as it can be a symptom of peritonitis. The force (*F)* necessary to make an indentation (d) in the abdominal wall is correlated with IAP as well as *C*_ab_:

5 $$F/d \, \approx \, IAP$$

**Fig. 5:**
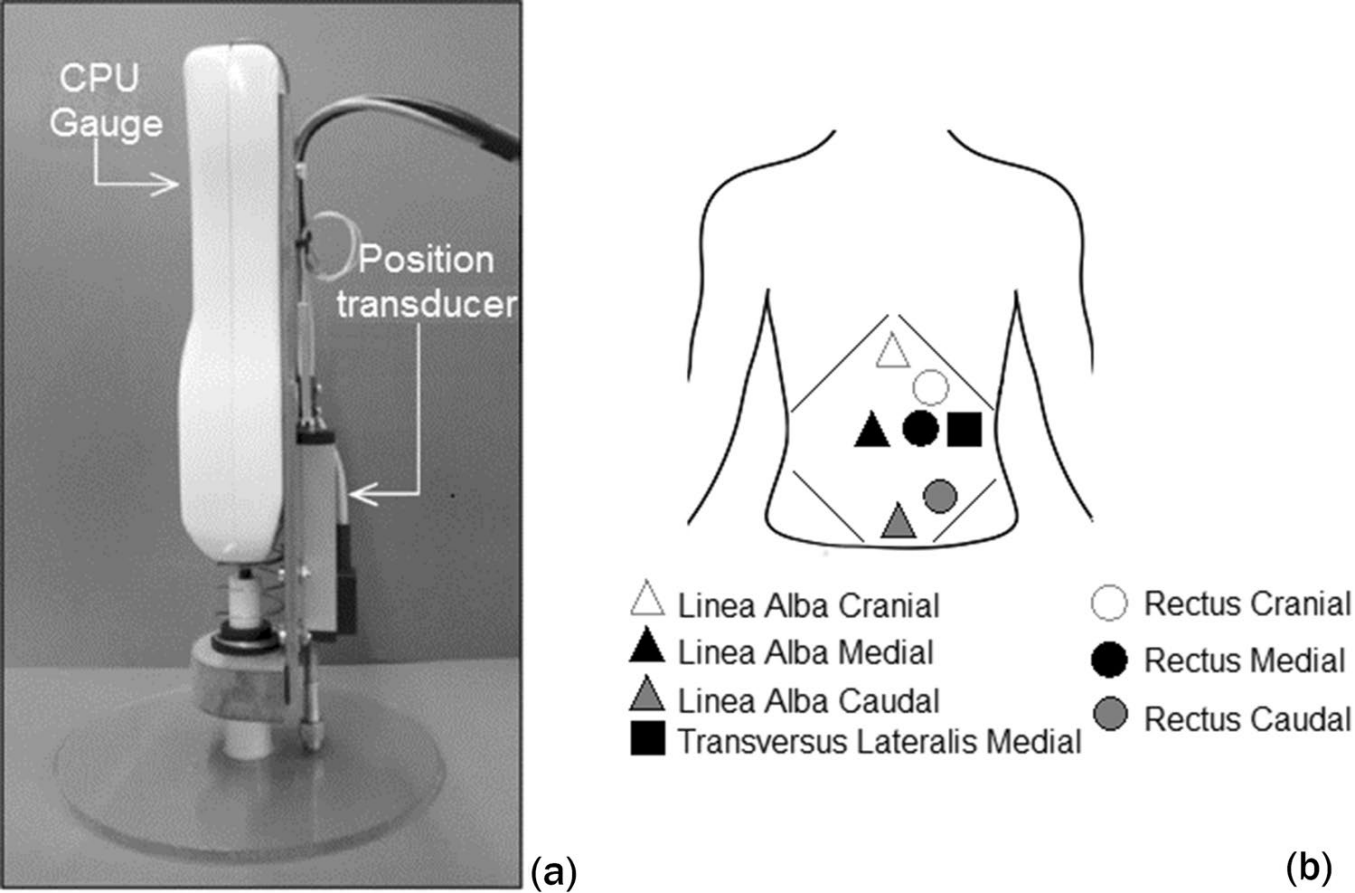
Abdominal wall tensiometry. Tensiometry is performed by measuring force and distance (indentation) at the site where the punctual force is applied (adapted with permission from van Ramshorst et al. [20]). **a** Initial abdominal wall tension (AWT) measurement device **b** Seven points were measured during the initial study: three on the linea alba, three on the rectus abdominis muscle and finally one over the lateral transverse muscle. The measurements were solely performed on one half of the abdomen, assuming abdominal symmetry

In a preliminary study, van Ramshorst et al. [20] examined abdominal wall tension (AWT) in 2 corpses (see Fig. 5 [20]). The abdominal cavity can be considered a cylindrical vessel (*t* < *R/4*; with *t* = abdominal wall thickness and *R* = radius). The tensile strength can thus be calculated by Eq. 6:

6 $$\sigma_{w} = \, [(P_{i} - \, P_{o} ) \, R]/t$$

Where σ_w_ is stress in abdominal wall (tension), *Pi* is internal pressure (IAP), and *Po* is external pressure.

In Fig. 5a [20], force and distance were registered simultaneously by using a CPU Gauge (Model RX Aikom, manufactured in Japan) and a position transducer (Series LWH, NovoTechnik, manufactured in Germany). Both sensors were supported by an assembly that enabled a device that applies a force, connected to the measuring end of the force meter, to pass through an acrylic foot. The zero point was set as the foot of the assembly and enabled the device to apply a force on the point of measurement, while the distant sensor measured the vertical displacement of the device [20].

**Fig. 6:**
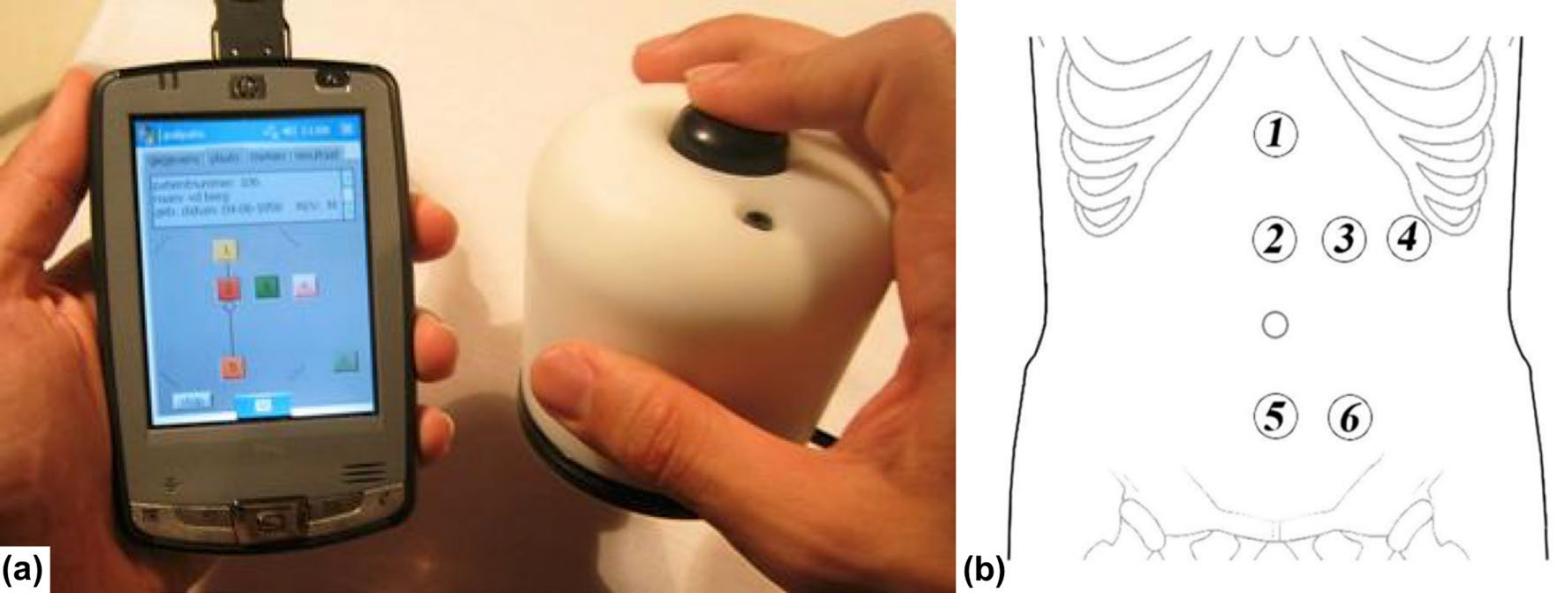
Abdominal wall tension (AWT) measurement prototype used by van Ramshorst et al. (adapted with permission from van Ramshorst et al. [21]). **a** New prototype of tensiometer connected to smartphone **b** Six measurement points, derived from anatomical structures, were marked on each abdominal wall: 5 cm caudal to the xiphoid bone (point 1), 5 cm cranial to the umbilicus (point 2), 5 cm left to point 2 (point 3), 10 cm left to point 2 (point 4), 5 cm cranial to the pubic bone (point 5), and an extra point, 5 cm left to point 5 (point 6)

In a later study, the same authors examined the abdomens of 14 corpses that were insufflated with air [21]. The IAP was measured at intervals of 20 mmHg. At each interval, abdominal wall tension (AWT) was measured five times at six different areas on the abdomen (see Fig. 6 [21]). In 42 volunteers, AWT was measured at five points in supine, sitting, and standing positions during various respiratory maneuvers. The authors found significant correlation between IAP and AWT (the best correlation was found in the epigastric region). In vivo measurements showed that AWT was on average 31% higher in men compared to women and increased from expiration to inspiration during valsalva maneuvers. AWT was highest in the standing position, followed by the supine and sitting positions. The body mass index (BMI) did not influence AWT.

Figure 6a [21] shows the prototype used for measuring AWT, consisting of a built-in force and distance sensor, attached to a handheld personal digital assistant (PDA, HP IPAQ). The diameter of the circle-shaped base of the device is 72 mm. The tip of the instrument is shaped like a half sphere and has a diameter of 18 mm, with a total surface area of 5.1cm^2^. The shape of the tip was chosen due to the extensive use of this shape in industrial hardness measurements of materials. The size of the tip was chosen due to its comparability to the human finger, which is used to palpate the abdominal wall. This device can measure the amount of force (N) needed to indent a specified amount (mm), which is then visualized on the personal digital assistant (PDA) with graphics [21].

**Fig. 7:**
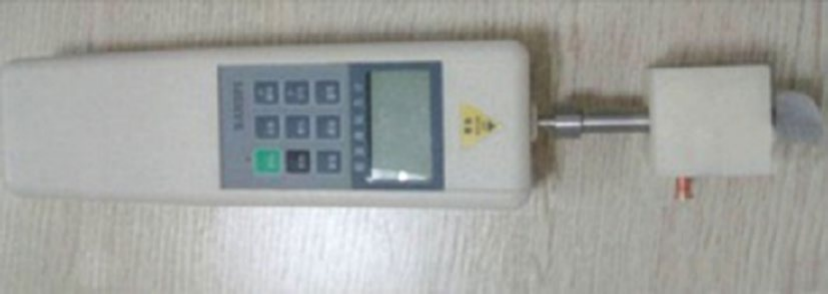
Abdominal wall tensiometer. Tensiometer used by Chen et al. to measure the required thrust (N) to produce displacement (mm) (adapted with permission from Chen et al. [22])

In another study by Chen et al. tensiometry was used as a non-invasive method for the assessment of urinary bladder pressure (UBP) [22]. This prospective study monitored 51 ICU patients with urethral catheters and studied the changes of AWT and UBP. UBP was in the range of 4 to 26 mmHg, and for each UBP value, the AWT was measured by a prototype AWT measurement instrument shown in Fig. 7 [22].

**Fig. 8:**
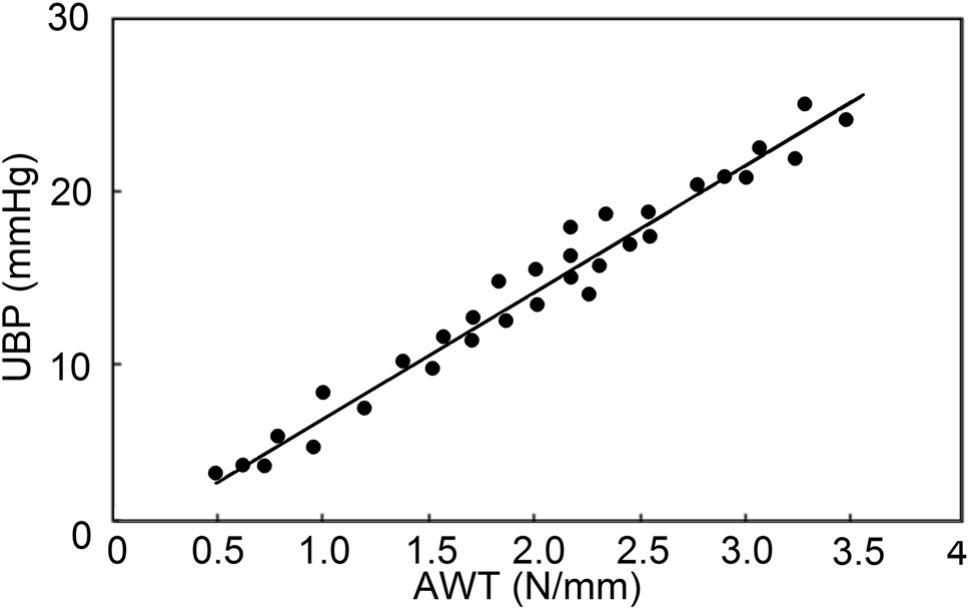
Correlation between abdominal wall tension (AWT) and urinary bladder pressure (UBP), performed by tensiometry. As can be seen, an almost linear correlation was found between abdominal wall thickness and the urinary bladder pressure (adapted from Chen et al. [22])

The tensiometer measured the required force to produce displacement. Thus, the AWT can be calculated as thrust/displacement (N/mm) [22]. The correlation between AWT and UBP is shown in Fig. 8 [22].

A significant linear correlation, with *R* = 0.986, *P* < 0.01, was found between AWT and UBP. Overall, it is feasible to use tensiometry for IAP measurement. It is fast and inexpensive with acceptable accuracy. However, as a new method, the AWT measurement devices should be standardized for a more reliable and reproducible assessment.

### Ultrasound-based techniques

Ultrasound is a common technique used in medicine. Healthcare professionals and medical companies are already familiar with the concept of using ultrasound-based devices to aid diagnosis. Furthermore, ultrasound is increasingly being used as a bedside modality in ICUs, referred to as point of care ultrasound (POCUS) [23].

There is limited research on ultrasound to measure IAP or to detect IAH/ACS. The possibilities of ultrasound as a measurement tool for IAP are explored in the following section.

#### Ultrasound tonometry

**Fig. 9:**
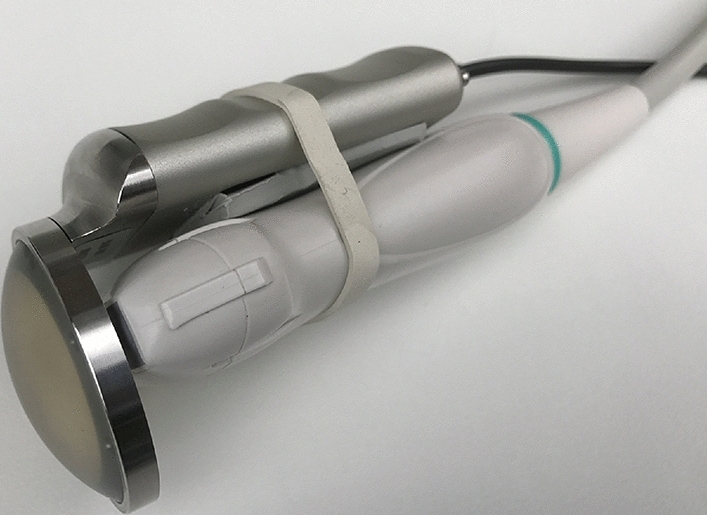
Veinpress system mounted on an ultrasound probe (adapted with permission from Bloch et al. [24])

Ultrasound in combination with tonometry (or ultrasound-guided tonometry) is not a particularly familiar concept in medicine. The principle is based on an ultrasound probe mounted onto an ultrasound transducer and connected to a pressure transducing system. This approach allows the sonographer to account for the pressure one exerts on the patient with the ultrasound probe. This technique was recently examined using the Veinpress 2014 system (Veinpress GmbH, 3110 Münsingen, Switzerland) as shown in Fig. 9 [24] (available for research purposes only). The system consists of an elastic silicone membrane filled with a fluid substance, translucent to ultrasound waves, and coupled to a manometer.

There are multiple approaches to measuring compartment pressure using ultrasound tonometry, however, this proposed approach is based on the research of Bloch et al. [24]. First, the elasticity ratio (ER) is calculated, which is the ratio of the compartment diameter with and without applying pressure. The pressure was in this case manually applied, but monitored by the Veinpress system.

**Fig. 10:**
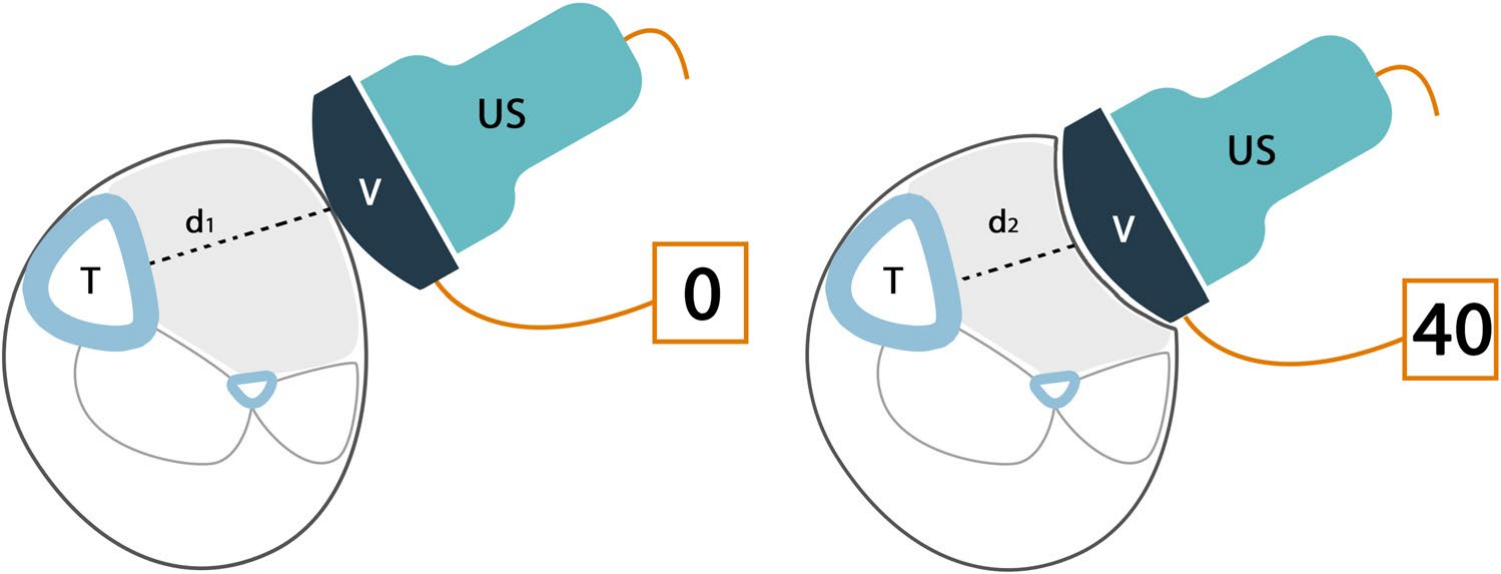
Ultrasound-tonometry set-up. Tonometry was performed in combination with ultrasound for intra-compartmental pressure (ICP) assessment with an applied pressure of 40 mmHg (right panel) compared to the situation without applied pressure (left panel) (d1, d2: compartment diameters, T: tibia, V: veinpress) (adapted from Bloch et al. [24])

**Fig. 11:**
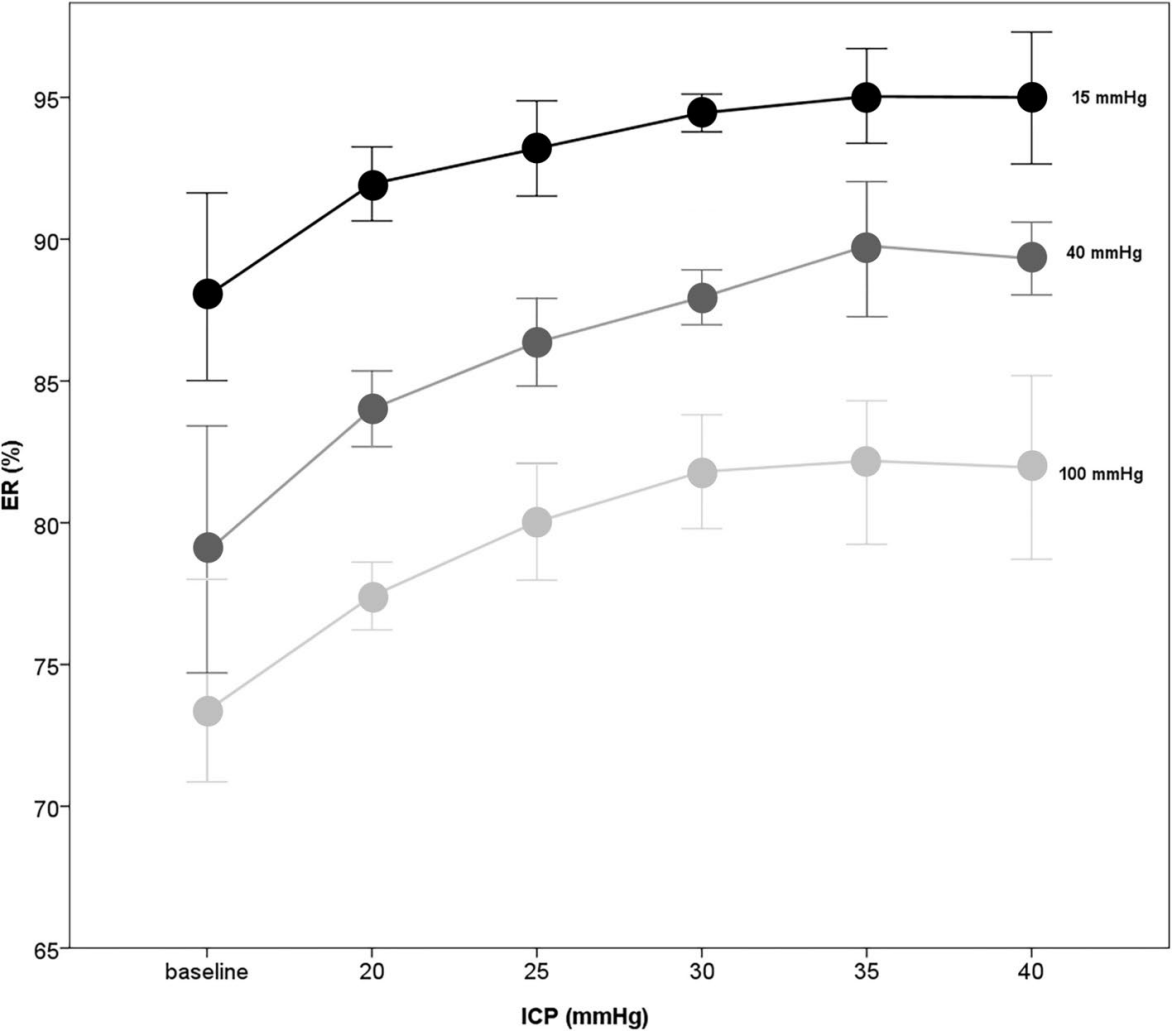
Elastic ratio (ER) as a function of the intra-compartmental pressure (ICP) for different external pressure values (adapted with permission from Bloch et al. [24])

The methodology of this experiment is shown in Fig. 10 [24], in which six porcine legs were used. The tibial compartment pressure was adjusted through a catheter that insufflated fluid. Although different external pressure levels of ultrasound can be used, results for an externally applied pressure of 40 mmHg (of ultrasound) showed the steepest ER increase (10.6%, from baseline to 40 mmHg vs. 7.0%– 9.7% for the respective others). Thus the most accurate differentiation of the intra-compartmental pressure (ICP) steps were achieved at 40 mmHg (see Fig. 11 [24]).

**Fig. 12:**
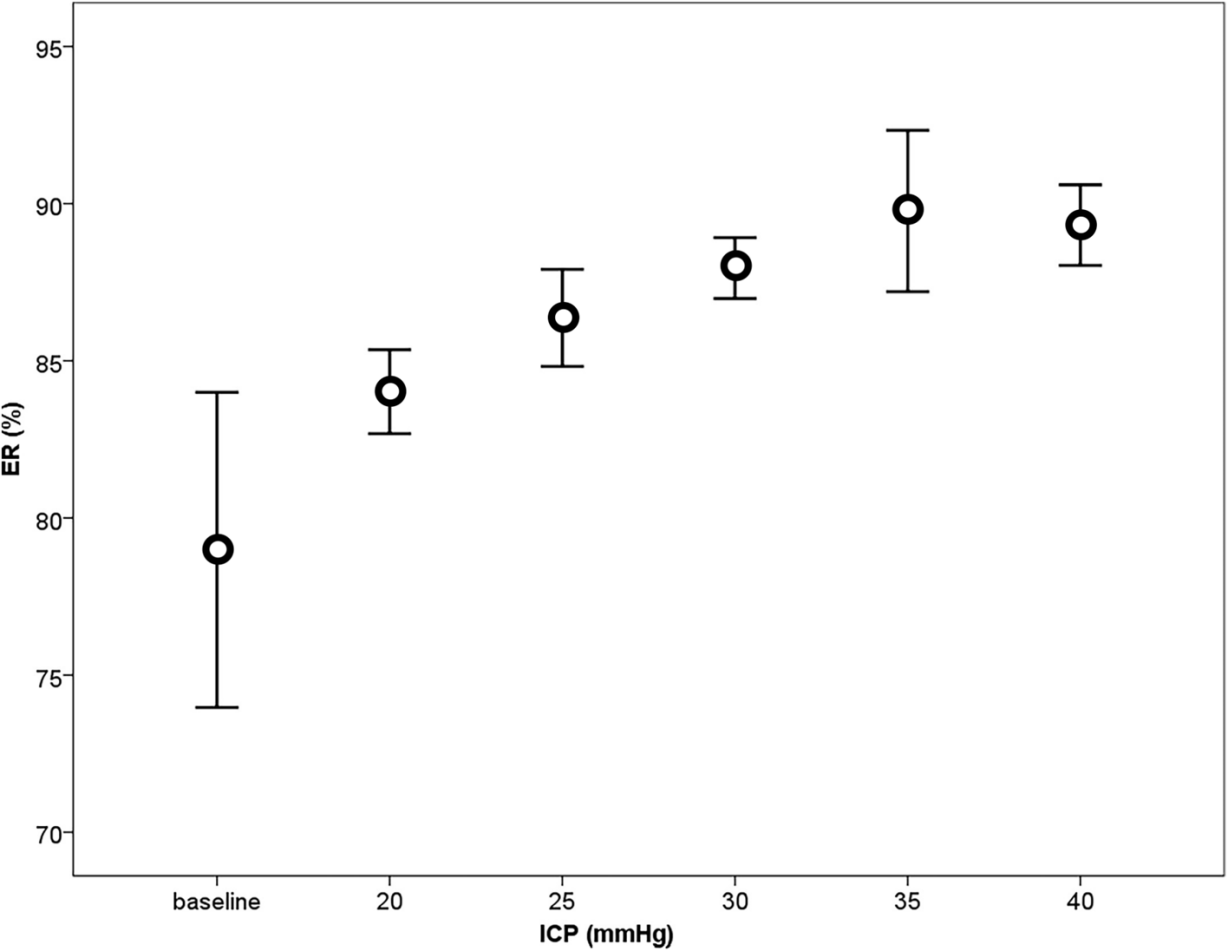
Elastic ratio (ER) as a function of the intra-compartmental pressure (ICP). The 95% confidence interval was seen at 40 mmHg external pressure (adapted with permission from Bloch et al. [24])

The resulting elastic ratios over a range of different ICPs are provided in more detail in Fig. 12 [24].

The results showed a correlation between the elastic ratio and the compartment pressure. An elastic ratio of 87.1% had a sensitivity of 94.3% to detect a pressure of 30 mmHg or higher [24]. However, as the pressure of the compartment rose above 30 mmHg, the ER tended to flatten. This may have resulted when a certain rigidity was reached and it became difficult to compress the tissue further.

The confidence intervals and the broad steps of 5 mmHg indicate that further improvements are required before this technology can be used as a bedside measurement device. The ultrasound tonometry could indicate hypertension in the tibial compartment, but does not suffice as a standalone measurement. Also, the diameter of a porcine leg is significantly smaller than the human abdominal cavity. The trade-off between depth of ultrasound penetration and resolution may make the measurement difficult in a large abdominal cavity. Additionally, the organs and layers of tissue in the abdominal cavity each have their own reflection coefficient. It is unclear if these measurements would be feasible in the case of increased IAP from different causes, and particularly difficult in the presence of abdominal gas.

**Fig. 13:**
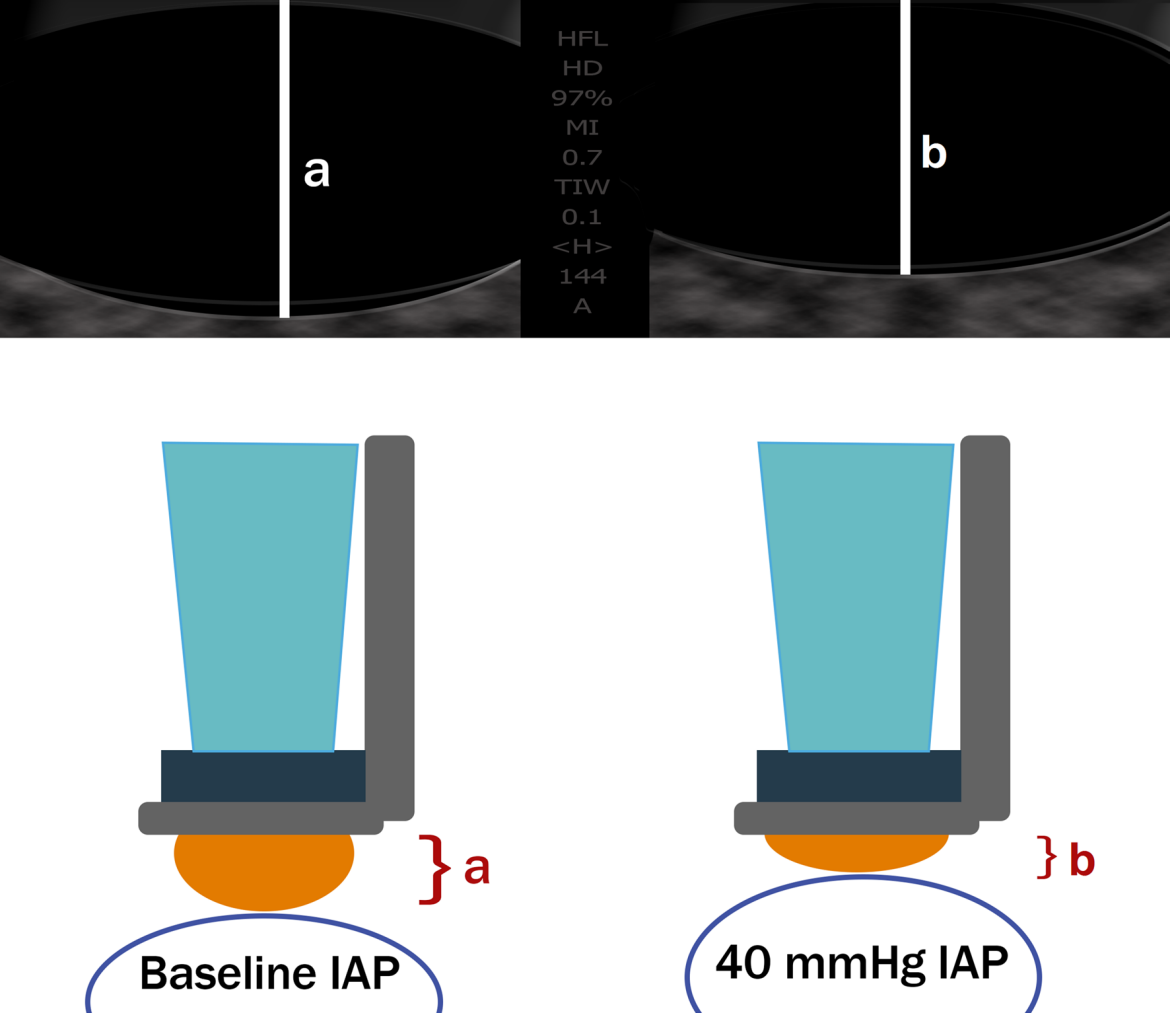
Ultrasound tonometry concept for intra-abdominal pressure assessment. Labels “a” and “b” denote the vertical chamber diameter in two different intra-abdominal pressure (IAP) values (adapted from Bloch et al. [25])

Bloch et al., focused on measuring IAP, hypothesizing that the vertical chamber diameter of the Veinpress system may inversely correlate with IAP [25]. The workflow of the experiment was similar to the previous one. A population of seven pigs was used and the IAP increased by instilling fluid into the abdominal cavity. Again, different pressure levels were induced (22.5 mmHg and 37.5 mmHg) while the chamber diameter was examined as a function of different stages of IAH (Fig. 13) [25].

**Fig. 14:**
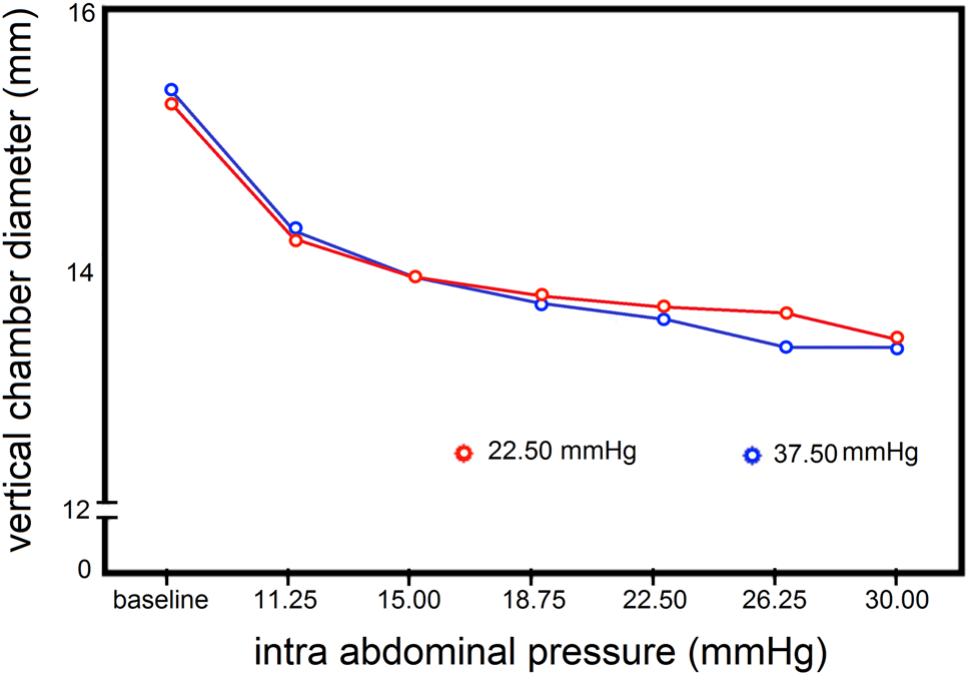
Vertical chamber diameter in relation to intra-abdominal prerssure. Chamber diameter is expressed as a function of intra-abdominal pressure (IAP) stages at an external pressure of 22.5 mmHg and 37.5 mmHg, assessed with ultrasound tonometry (adapted from Bloch et al. [25])

The results (Fig. 14) [25] showed a correlation between the IAP and the ultrasound tonometry measurements [25]. The system was only able to discriminate between three pressure ranges, namely between baseline and 15 mmHg, between 15 and 25 mmHg, and between 25 and 40 mmHg (p-value < 0.02) [25]. All other ranges showed overlap and indicated very poor accuracy. Knowing that IAP is only elevated when > 12 mmHg, and ACS generally occurs above 20 mmHg, this device may only offer an indication of increased IAP (grade 2 and higher) without the ability to discriminate higher grades of IAH. A more reliable and accurate clinical measurement is required.

Ultrasound tonometry has advantages in that it is non-invasive and easily reproducible, requires only minor changes to a classic ultrasound examination, and is relatively inexpensive. It may also appeal to medical personnel, in that it is a safe and familiar modality. However, the need for coupling gel during an ultrasound examination limits this technology when adapting it to be a continuous process.

The major disadvantage of this technology is most notably its lack of accuracy. However, the majority of research dates from 2017, and additional research may provide solutions.

#### Ultrasound assessment of the abdominal wall in combination with external pressure

See et al. proposed an alternative ultrasound-based IAP measurement technique [26]. A correlation was found between IAP measurement using this ultrasound technology and IAP measured by the standard intravesical method. Two operators performed the ultrasonography and a third operator used the intravesical method of IAP measurement.

**Fig. 15:**
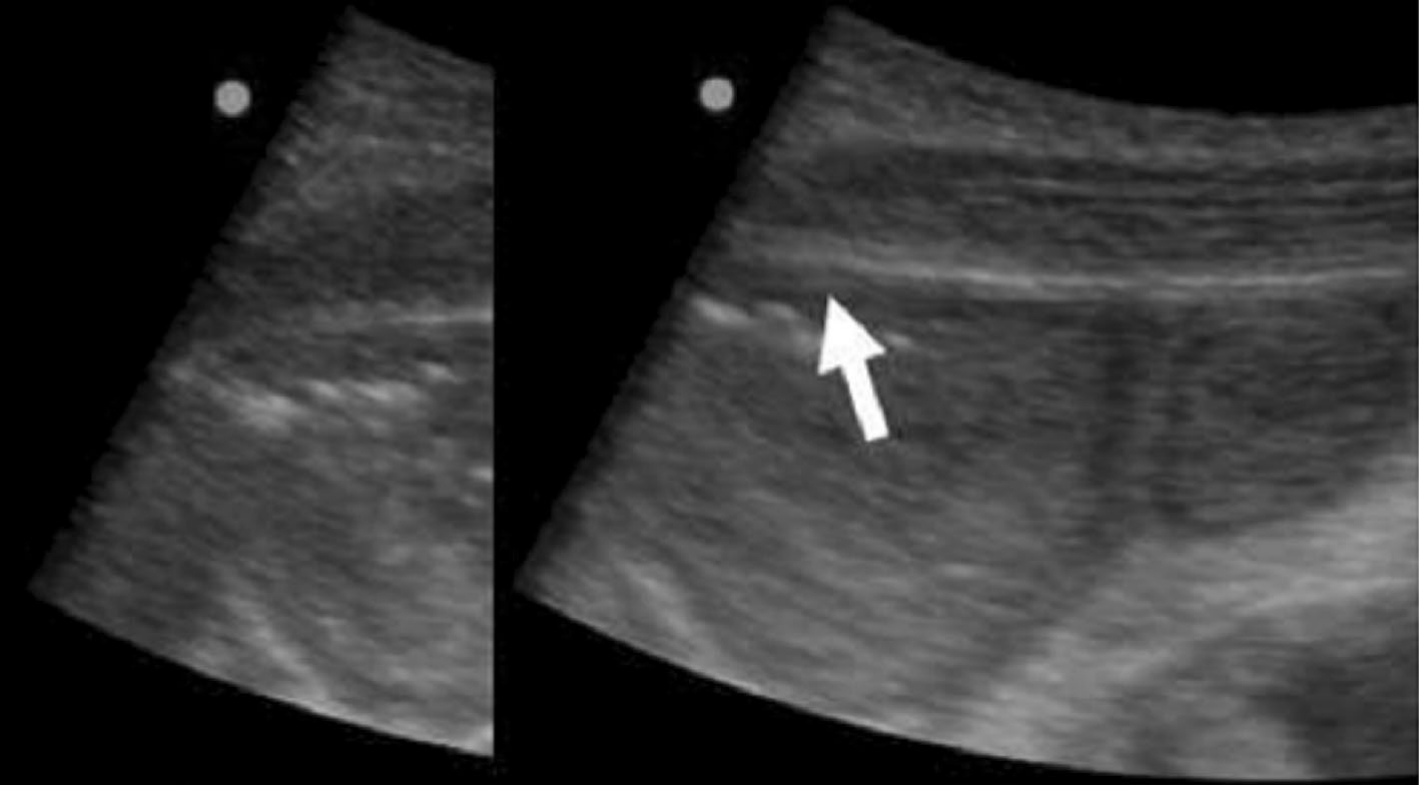
Peritoneal rebound visualized by ultrasound assessment in combination with external pressure. Loss of peritoneal rebound visualization happens when IAP is equal to or more than external pressure (adapted with permission from See et al. [26])

A bottle filled with decreasing amounts of water was used to apply a decremental pressure on the abdominal wall, while ultrasound was used to determine the peritoneal rebound (see Fig. 15 [26]). The concept is based on the loss of peritoneal rebound being a sign that the IAP is equal to or more than the external pressure. In Fig. 15 [25], peritoneal rebound is seen, indicating that the IAP is less than the external pressure.

The results from the ultrasound operators showed a very good correlation with the current gold standard for IAP measurement (R = 0.982 and p < 0.001) and could be a clinically reliable non-invasive technique in the future [26].

#### Ultrasound Doppler tonometry

**Fig. 16:**
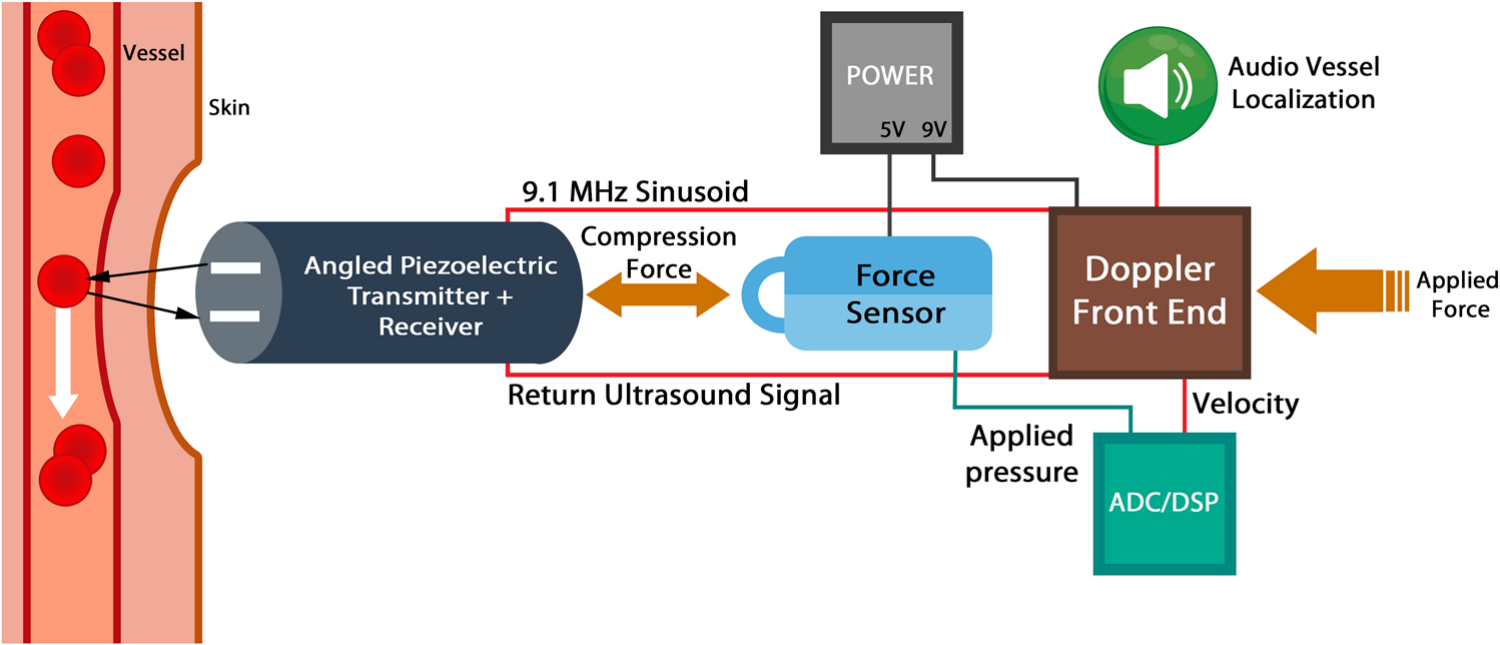
Block diagram of a Doppler tonometry system (adapted with permission from Akinin et al. [27])

Doppler ultrasound is a non-invasive technique that measures flow through blood vessels, by making use of the frequency shift of soundwaves reflecting off moving red blood cells. The frequency change is in proportion to the blood flow velocity. However, classical doppler ultrasound faces some challenges, most notably the velocity waveform is affected by the pressure applied by the operator. The ultrasound probe needs to be firmly in contact with the gel and skin surface, and maintaining this appropriate constant pressure by the operator is difficult. Doppler tonometry was introduced in an attempt to help overcome this problem. A force sensor is introduced to feedback any pressure information. Figure 16 [27] presents a block diagram of the basic components of the doppler tonometry system.

**Fig. 17:**
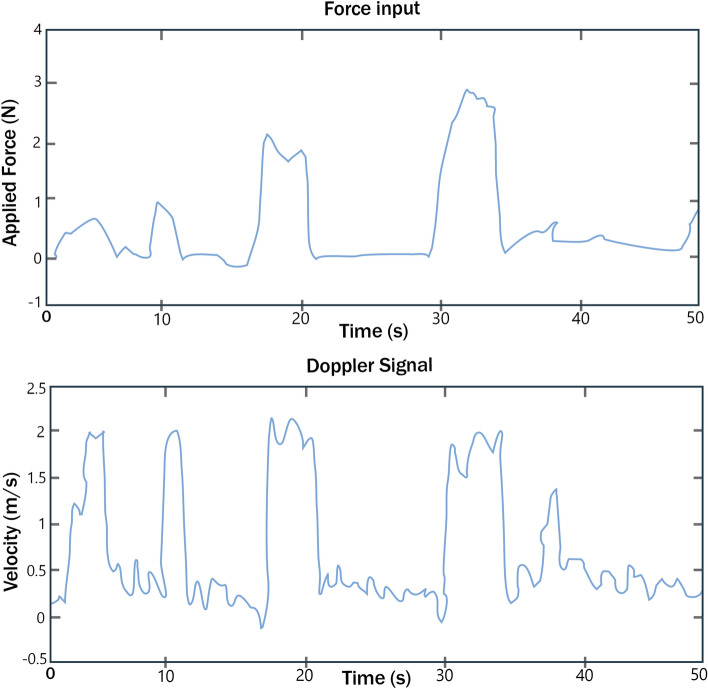
Relationship between applied force and measured velocity as a function of time (adapted with permission from Akinin et al. [27])

Akinin et al. designed and manufactured a Doppler tonometer sensor system with a sub-resolution of 0.1 N. Multiple tests were performed including a study on a subject's arm [27]. A vein was located and cycles of increased and decreased pressure were implemented. Figure 17 [27] shows the applied force and corresponding velocity as a function of time.

The graph shows that a relatively small force had a significant effect on velocity, largely because veins, more so than arteries, are susceptible to deformation. By decreasing the cross-sectional area of a vessel, the velocity was increased. Including force feedback could significantly improve the reliability of this measurement.

**Fig. 18:**
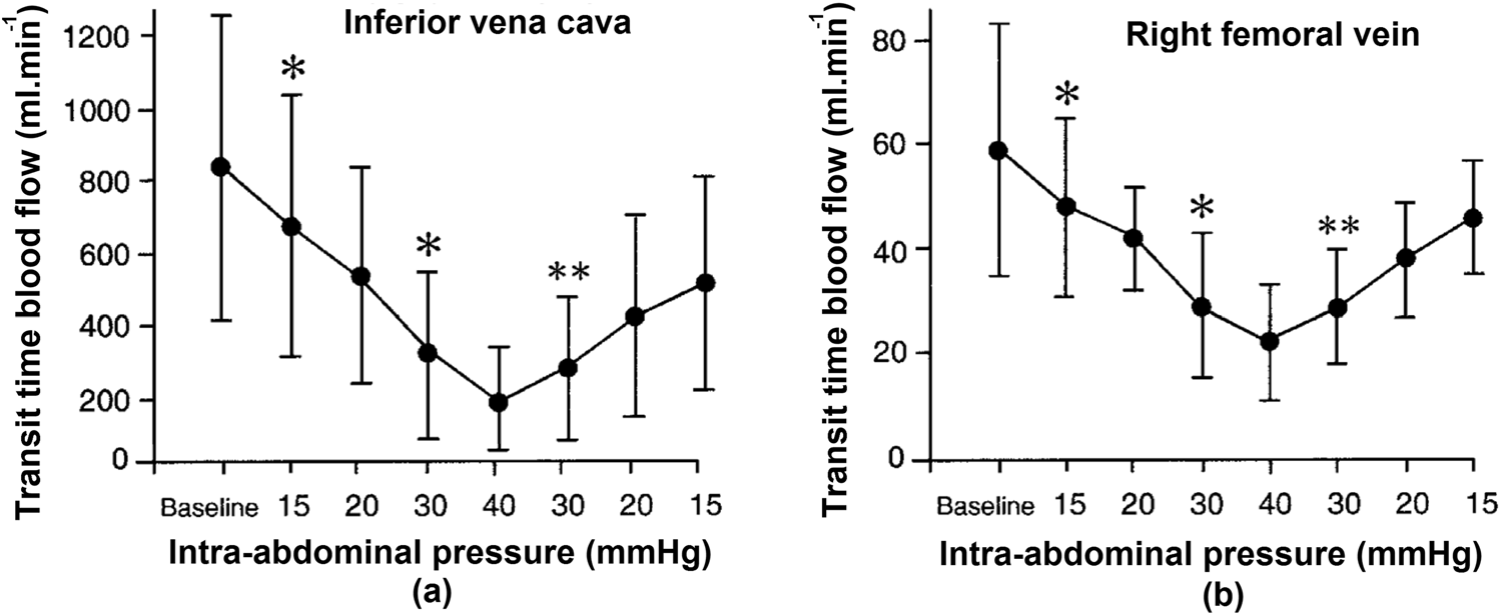
Doppler ultrasound. Relation between intra-abdominal pressure (IAP) and venous transit time blood flow, performed by Doppler ultrasound. (adapted with permission from Gudmundsson et al. [28]). **a** Blood flow in the inferior vena cava vein and blood flow in the right femoral vein **b** as a function of intra-abdominal pressure. * significant difference from the nearest left observation, ** significant difference from the final IAP measurement (P < 0.05)

The idea of using ultrasound Doppler tonometry to measure IAP came from the theory that a correlation exists between blood flow in certain veins and the IAP. Gudmundsson et al. investigated this further [28] through inducing IAP on eight porcine subjects. Blood flow measurements were taken by transit-time flowmetry (Transonic Systems, HT 107) (Fig. 18) [28].

The objective was to investigate the correlation between inferior vena cava/right femoral vein flow and IAP. The results showed a significant decrease in blood flow when IAP increased, and this was similar in both veins [28]. However, this method was inaccurate and only major changes in IAP were detected. Furthermore, there is no data on measuring blood velocity as a function of IAP with a Doppler sound tonometer sensor system. The system used by Gudmundsson et al. was a transit time flowmeter that was solely used on animals [28]. Unfortunately, there is a lack of data from further studies. Moreover, as an indirect measurement of IAP, this method does not surpass the accuracy of ultrasound tonometry technology.

#### Laser-ultrasound

Multiple problems arise when using ultrasound techniques to measure IAP. Laser-ultrasound was introduced to overcome the lack of accuracy and the problem of needing to use coupling gel. The photoacoustic working principle of laser-ultrasound is based on the optical excitement of tissue with a laser pulse. The absorbed light is converted into heat and introduces temporal thermo-elastic pressure-waves. An ultrasound receiver then detects these pressure waves. The receiver in this case is not a typical piezo-electric transducer, but an optical microphone. Preißer et al. proposed a commercially available optical hydrophone from XARION Laser Acoustics GmbH (Vienna, Austria) [29]. However, for IAP measurement, a membrane free optical microphone is more suited.

**Fig. 19:**
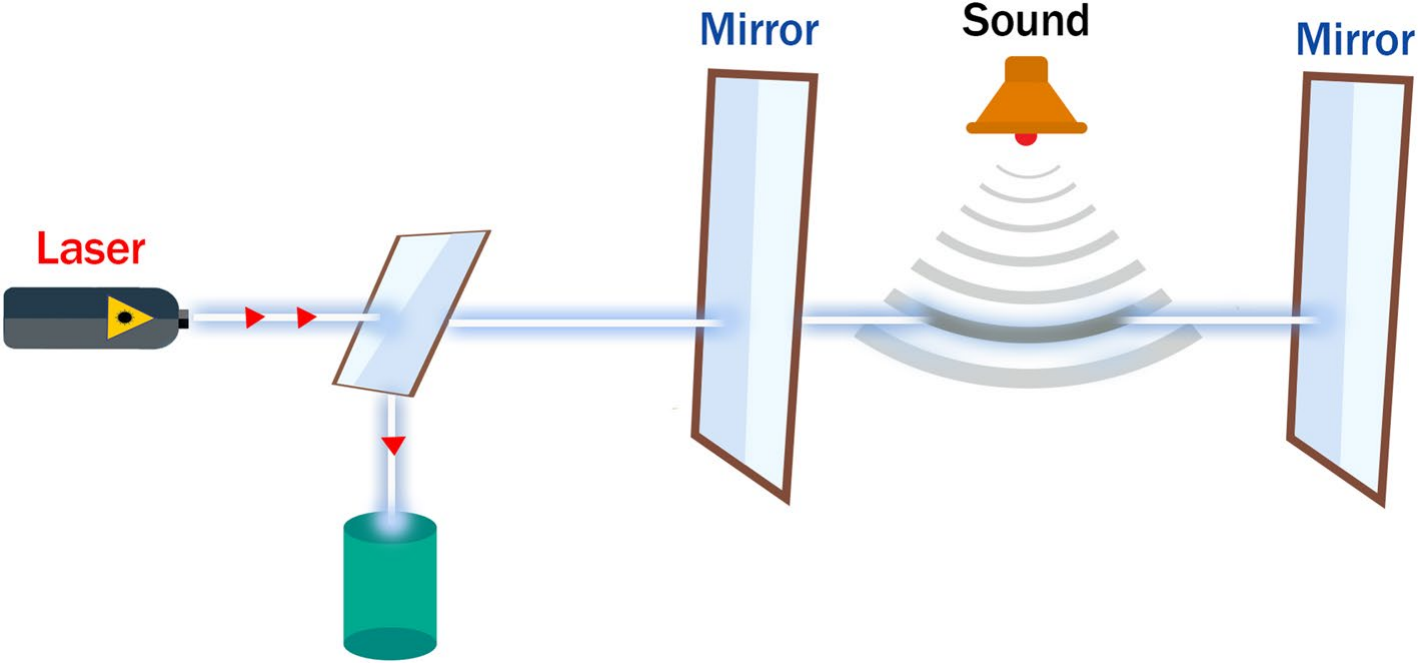
Laser ultrasound. Schematic representation of the optical sensor and Fabry-Pérot etalon as the main concept of laser ultrasound (adapted with permission from Preisser et al. [29])

The theory involves the ultrasound pressure wave inducing a density change in the medium of the optical sensor [29]. These changes are detected by a so-called Fabry-Pérot etalon, which is schematically shown in Fig. 19 [29] and, in essence, is a compressed laser interferometer.

The ultrasound waves cause a density change, which in turn cause a change in the refractive index of the mirrors. A laser sensor present in the etalon has its wavelength altered due to this phenomenon and, therefore, the ratio of the reflected light is increased. This effect is measured by a photodiode [29].

This method could be implemented for IAP measurement. The laser pulse could be introduced anteriorly and measured posteriorly on the patient. When IAP rises, the pressure wave moving through the body will be altered introducing a change in the received signal (in dB). However, attenuation due to different tissues must be considered. Pressure waves would travel back and forth through the numerous layers in the body, and attenuation of these waves could have a serious impact. It is unclear how this signal would be received.

Another approach would be to place both the emitter and receiver anteriorly. The elastic properties of underlying tissue would be altered by the increase in IAP and this change would be picked up by the laser-ultrasound receiver. To our knowledge no such experiments have yet been carried out for measurement of IAP.

Laser-based ultrasound offers multiple advantages including higher sensitivity compared to classic ultrasound techniques, no need for coupling gel or direct contact, and no clamping or pressure forces required. Moreover, it operates in a vast dynamic range and offers good mechanical stability. However, there is no published evidence of proof of concept yet, and this cannot be crowned as the ultimate solution.

### Bio-electrical impedance and microwave reflectometry

#### Bio-electrical impedance

**Fig. 20:**
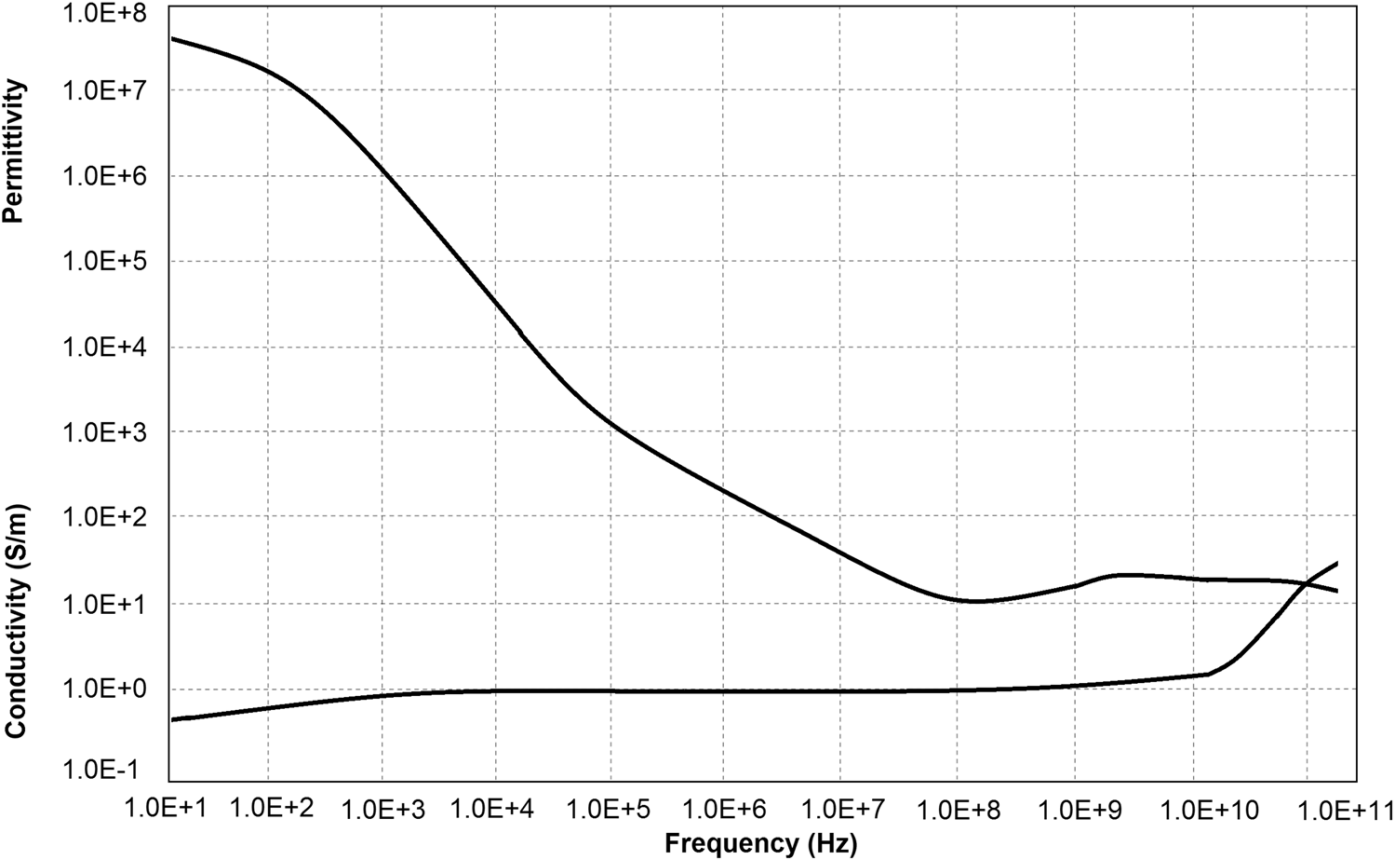
Experimental data for permittivity and conductivity of muscle at body temperature (adapted with permission from Gabriel et al. [30])

Electrical conductivity and permittivity are generic properties common to all materials. They define the in-phase and in-quadrature response of any material to applied AC (alternating current) and DC (direct current) electrical fields. Both material properties are tissue-specific (see Fig. 20 [30]) and can be determined by applying low-level AC and DC voltages and measuring the current (density) in phase and in quadrature, which is related to the complex-valued electrical impedance. For biological materials, one refers to the bio-impedance characterization of tissues and organs. Bio-impedance measurements can be considered as a non-destructive and non-invasive measurement technique. The study of the electromagnetic properties of specific body tissues can yield insights into physiological functioning and can therefore be used for disease correlations, such as IAP.

David et al. published a pilot study where they performed non-invasive measurement of IAP using bioimpedance in a porcine model [32]. It was assumed that changes in IAP were directly correlated to the abdominal wall thickness. The abdominal wall was seen as a compound of parallel layers where the change in thickness of these layers affected the measured bioimpedance values, thus giving a value to the IAP.

The abdominal wall was modeled as a compound of five tissue layers [32]:

1. Peritoneum
2. Muscles, including internal and external obliques, and transversus abdominis
3. Fascia divided in Camper’s (fatty) and Scarpa’s (fibrous) fascia
4. Fascia transversalis
5. Skin and subcutaneous tissue

The different layers of the abdominal wall were characterized by their overall dielectric permittivity function ${\varepsilon }_{T}^{*}\left(\omega \right)$.

The dielectric permittivity of each layer mentioned above were calculated by Eq. (7):

7 $$\varepsilon_{n}^{*} \left( \omega \right) = \varepsilon_{n} \left( \omega \right) + \frac{\sigma \left( \omega \right)}{{j\omega \varepsilon_{0} }}$$

where ${\upvarepsilon }_{0}$, $\upsigma$, and ${\upvarepsilon }_{\mathrm{n}}$ are the permittivity of vacuum, conductivity and relative permittivity studied by Gabriel et al. [30].

The overall dielectric function for a structure consisting of two layers is modeled in Eq. 8 [31].

8 $$\varepsilon_{T}^{*} \left( \omega \right) = \left( {d_{1} + d_{2} } \right)\cdot\frac{{\varepsilon_{1}^{*} \left( \omega \right)\varepsilon_{2}^{*} \left( \omega \right)}}{{d_{2} \varepsilon_{1}^{*} \left( \omega \right) + d_{1} \varepsilon_{2}^{*} \left( \omega \right)}}$$

The factors $\omega$, ${\varepsilon }_{1}^{*}$, ${d}_{1}$, ${\varepsilon }_{2}^{*}$ and ${d}_{2}$ are the frequency, dielectric permittivity, and thickness of each layer, respectively. Although Eq. 8 is for a structure consisting of two layers, the general template of the equation is the same for structure consisting of more layers.

As a result, the overall complex-valued electrical impedance measured over the abdominal wall was a function of the complex permittivity of each layer inside the multi-layer structure. Due to the change in IAP and the elastic properties of each layer, the multi-layer structure was strained and thickness variations were induced. Finally, the induced thickness variations were correlated with IAP changes.

**Fig. 21:**
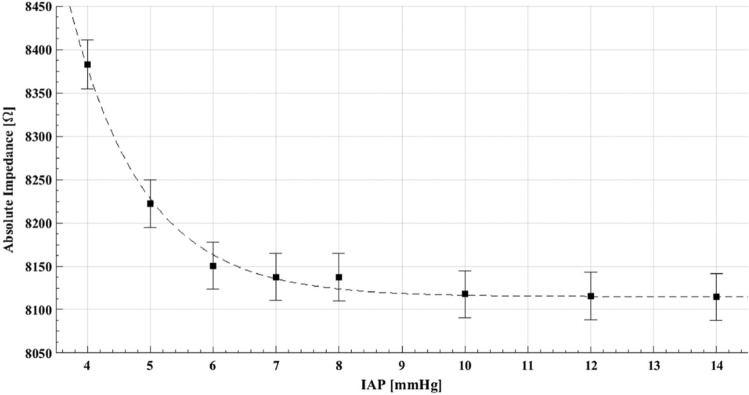
Bioimpedance analysis. Results of the measured absolute impedance for different induced values of IAP. Drastically reduced sensitivity for values over 7 mmHg is observed (adapted with permission from David et al. [31])

Bioimpedance has been previously used in a variety of areas [32]. There was a strong correlation between IAP and absolute impedance for IAP up to 7 mmHg (see Fig. 21 [31]). Although bioimpedance can be used as a completely non-invasive measurement technique for IAP levels up to 7 mmHg, further research is needed to validate the sensitivity of this method for higher pressures, before it becomes clinically useful.

#### Microwave reflection

Microwaves are non-continuous electromagnetic waves in the frequency spectrum of 300 MHz and 300 GHz. Microwave technology is widely used, with applications such as wireless networks, radar, remote sensing and controlling, and microwave ovens. Some advantages of microwaves are the larger bandwidth compared to lower frequency radio waves and better directive properties. This makes microwave high gain antennas easier to design and manufacture, and the use of higher frequencies allows for a decrease in the size of such antennas. Microwaves can be safely used in medicine for both diagnosis and treatment strategies. Several applications have been developed for microwave characterization of biological materials at molecular, cellular and tissue levels [33]. Some examples include the diagnosis of malignant tumors, real-time body fluid analysis, and therapeutic and surgical applications. A widely developed healthcare application of radiofrequency and microwaves is magnetic resonance imaging (MRI), in which signal information is used for reconstructing MRI images.

In the same study discussed previously in the bioimpedance section, David et al. [31] studied the non-invasive assessment of IAP using electromagnetic waves in the microwave range, in particular at 4.25 GHz. Using the same five-layered model of the abdominal wall and assuming that the IAP ultimately alters the thickness and electromagnetic properties (permittivity and conductivity) of the abdominal wall, the reflection response to incident electromagnetic waves was assumed to be different. In this study [31], porcine subjects were studied with different IAP values.

When working with frequencies of a few GHz, the layered anatomy was assumed to be a structure of lossy dielectric slabs. The reflection response for each interface was calculated by Eqs. (9) and (10) [31]:

9 $$\Gamma_{i} = \frac{{\rho_{i} + \Gamma_{i + 1} e^{{ - 2jk_{i} l_{i} }} }}{{1 + \rho_{i} \Gamma_{i + 1} e^{{ - 2jk_{i} l_{i} }} }}$$

10 $$\rho_{i} = \frac{{\eta_{i} - \eta_{i - 1} }}{{\eta_{i} + \eta_{i - 1} }}$$

Factors ${\Gamma }_{i}$, ${\rho }_{i}$, ${\eta }_{i}$, ${k}_{i}$ and ${l}_{i}$ represent the layer reflection response, primary interface reflection coefficient, characteristic impedance, angular wavenumber and the thickness of the ${i}^{th}$ slab, respectively.

As a result, changes of IAP will change the thickness of each layer of the abdominal wall, and hence the reflection response from the abdominal wall will be a function of the IAP [31].

**Fig. 22:**
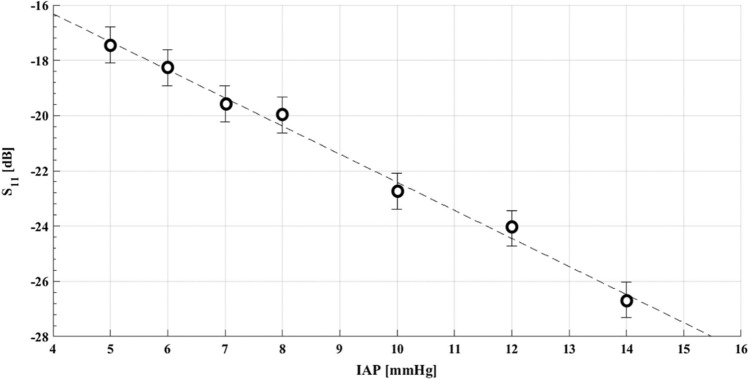
Microwave reflectometry. Values of S11 (scattering parameter) as a function of IAP for the selected frequency of 4.25 GHz, measured based on microwave reflectometry (adapted with permission from David et al. [31])

The results of the study show a strong correlation between IAP and the scattering parameter S_11_, corresponding to the reflection coefficient of the multi-layer structure, for the entire range of IAP measured (see Fig. 22 [31]). This was different to the results of the same study for bioimpedance, which only showed correlation up until 7 mmHg. David et al. [31] thus demonstrated that microwave reflectometry measurement technique can be used to measure IAP. It is continuous, non-invasive and sensitive enough to changes in IAP. However, the quality of the measurement can be easily affected. Further validation is required for this measurement technique.

### Digital image correlation and wireless motility capsule

#### Digital image correlation

**Fig. 23:**
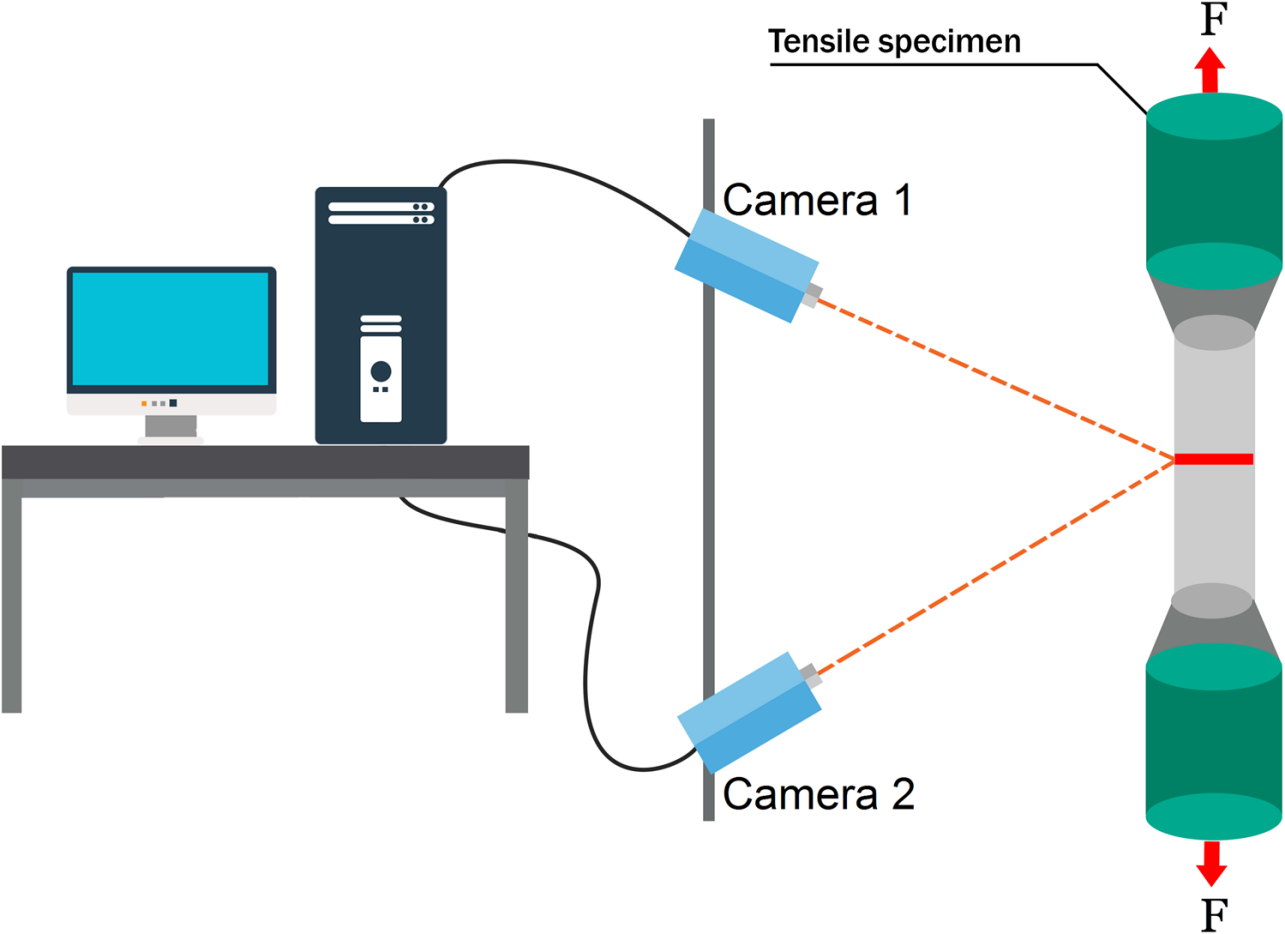
Digital image correlation. Set-up of digital image correlation (DIC) test for a uniaxial tensile test of a cylindrical specimen. (adapted with permission from [34])

Digital image correlation (DIC) is an optical method that uses tracking and image registration to obtain accurate measurements of contour, deformation, and strain on almost any material. An unambiguous speckle pattern is sprayed on the area of interest for tracking. The working principle is based on the localization of specific pixels using a stereoscopic sensor (camera) on each point of the object. A displacement field is then obtained from the correlation between consecutive images based on the random speckle pattern. With this data, the three-dimension position (or two-dimension position) of the object can be calculated. Applying the correlation algorithm can identify the position of each point of the object. For effective measurements, the pixels should be easily recognizable and requires a special illumination technique. Figure 23 [34] illustrates a typical set-up for DIC in the case of a uniaxial tensile test specimen.

**Fig. 24:**
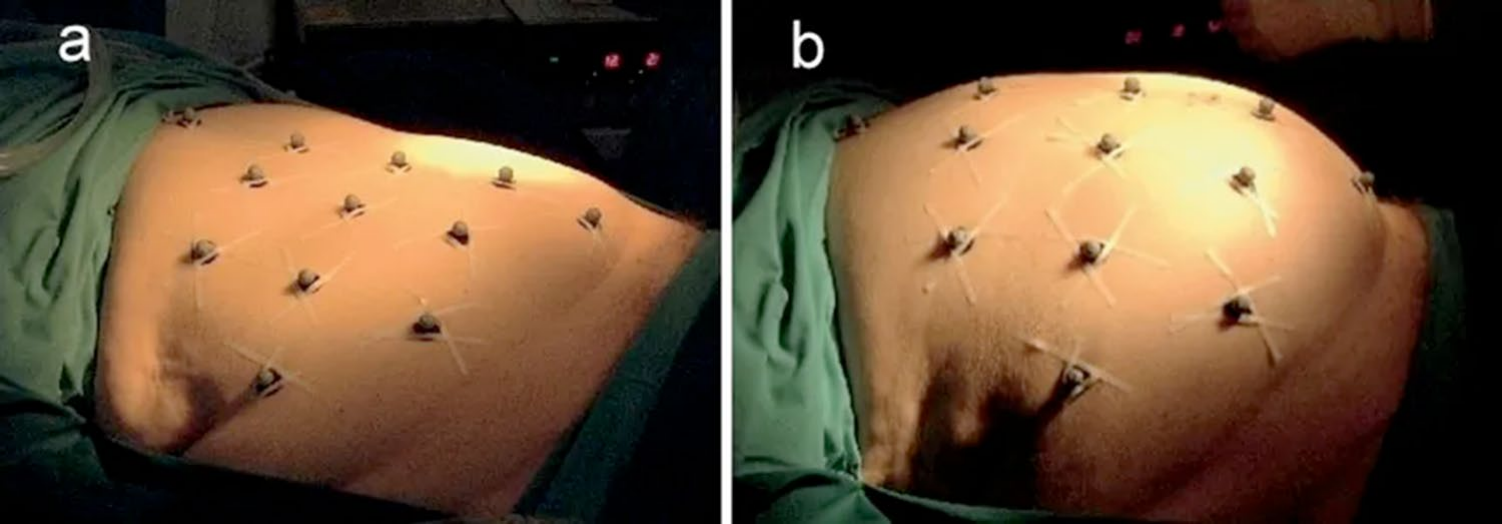
Markers placement on the abdominal skin for digital image correlation **a** Markers configuration in normal IAP **b** Markers configuration in IAH (adapted with permission from Song et al. [35])

Although digital image correlation has not been used for IAP measurement, Song et al. [35] used a similar concept to investigate the mechanical properties of abdominal wall during insufflation (see Fig. 24 [35]).

DIC is an accurate, but expensive technique. Moreover, keeping a random pattern placed around the patient's abdomen intact and visible, with an optimal view of the surface for the camera, is not practical. Another troublesome aspect of performing DIC in the ICU is the space required for the cameras. Positioning cameras from the ceiling is a possibility, but there should still be easy access to the camera system for calibration, troubleshooting, and maintenance. The accuracy of this technology is the greatest advantage, however, this does not outweigh the cost and additional problems already highlighted.

#### Wireless motility capsule

Using smart pills seems a useful method for non-invasive measurements of IAP. Rauch et al. evaluated IAP in a porcine model with a wireless motility capsule [36].

**Fig. 25:**
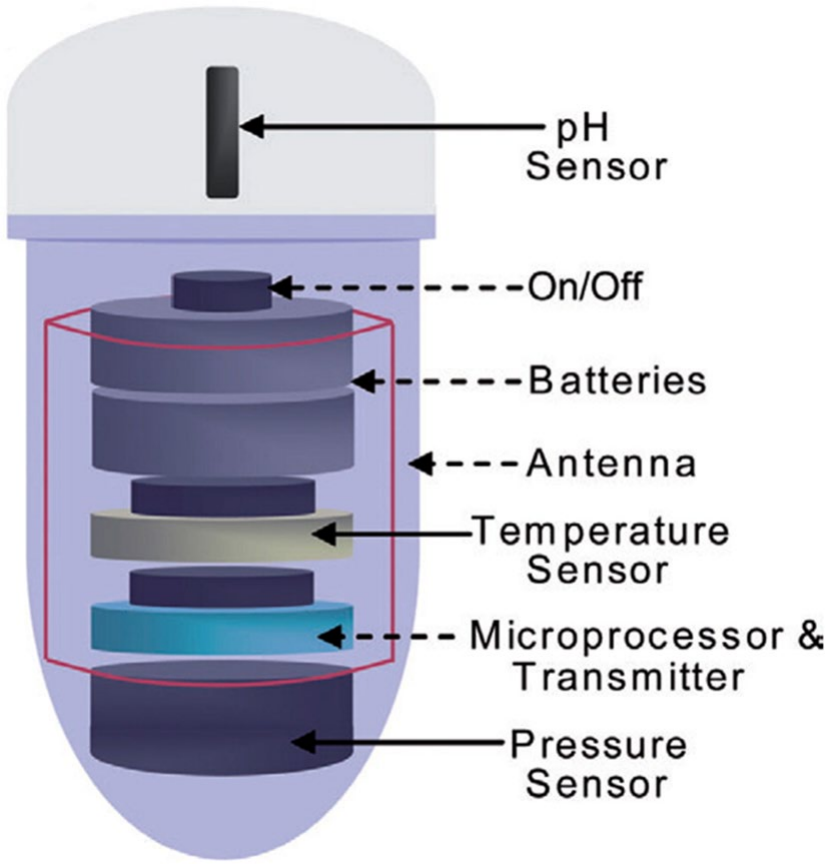
Wireless motility capsule. Components of a motility capsule for intragastric pressure measurement consists of a solid plastic head and a soft polyurethane body incorporating the batteries and sensors (adapted with permission from Fernandes et al. [37])

**Fig. 26:**
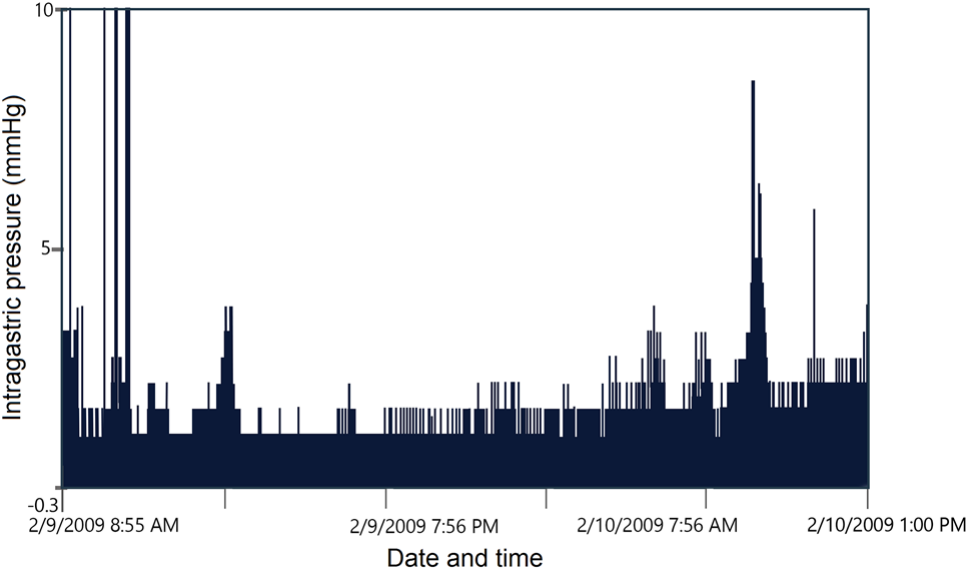
Intragastric pressure changes in an individual pig model during 24 h measured by wireless motility capsule (adapted from Rauch et al. [36])

Generally, a motility capsule (Fig. 25) [36] is a wireless smart pill that transmits pH, pressure, and temperature data to a data recorder [35]. In this study, by means of a capsule delivery device, a developed motility capsule (SmartPill™, SmartPill Corp., Buffalo, NY) was positioned endoscopically to the stomach of a porcine model to measure the intra-gastric pressure. In Fig. 26 [36], intragastric pressure values recorded by the motility capsule can be seen. In the end, the results from the capsule were compared to the pressure values achieved by intravesical method to study the correlation between these two different techniques.

This study reported an underestimation of IAP when using the motility capsule and measuring intragastic pressure [36]. The discrepancies between the two methods could be caused by gastric dilatation [36]. The poor correlation between intragastric and intravesical pressures seems to be due to the location and not the measurement technique [36].

We believe further research should be done regarding this technique in order to critically evaluate the reliability of motility capsules in the detection of IAH. Since there is no study dealing with insufflation of the abdominal compartment and monitoring IAP changes by motility capsules, we cannot comment on the usefulness of this device. It may be considered as a potential IAP assessment tool in the future.

## Summary and conclusions

**Fig. 27:**
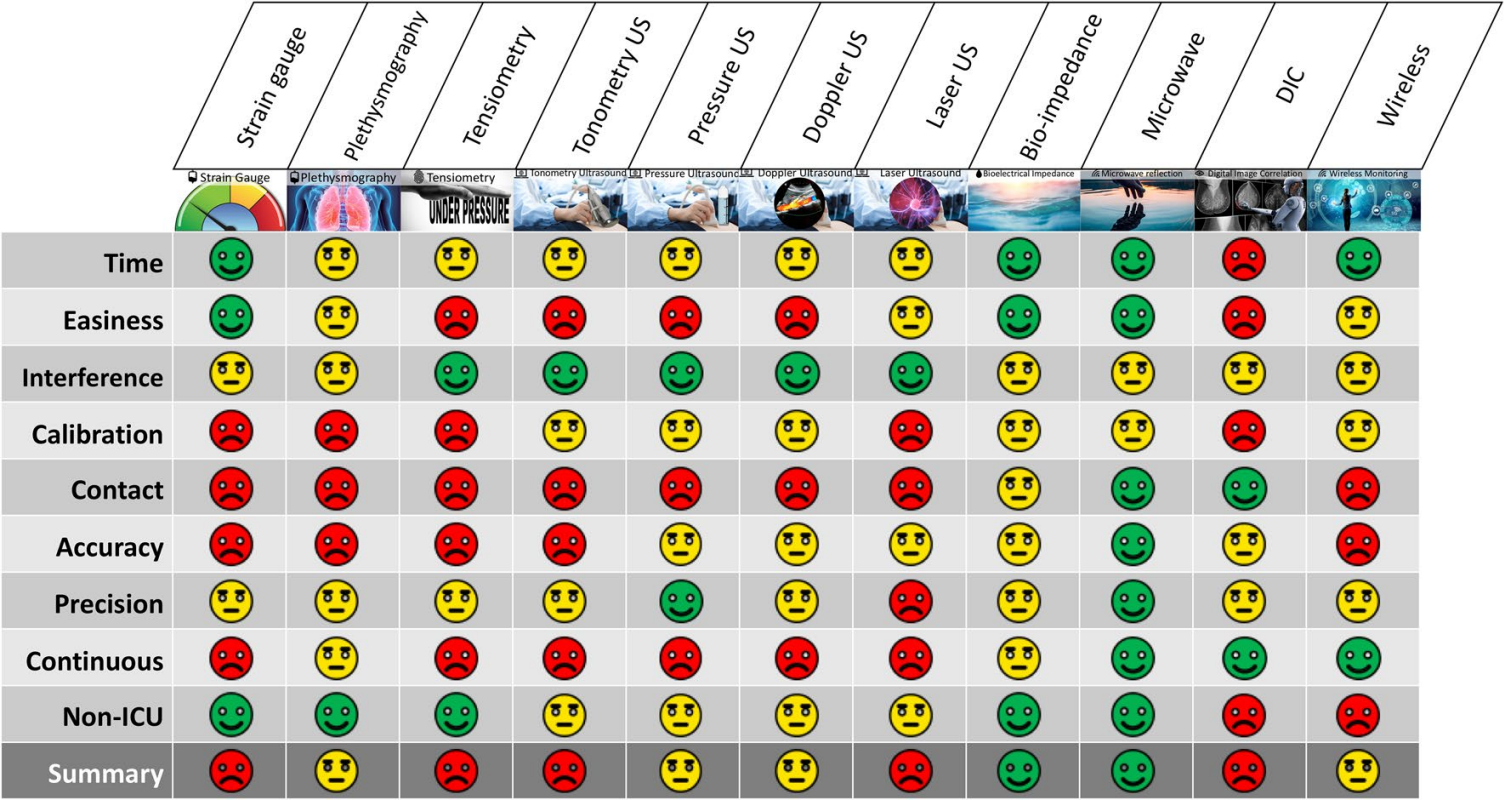
Summary of characteristics of the different non-invasive IAP measurement techniques. (DIC: digital image correlation, US: ultrasound)

The advantages and disadvantages of the described non-invasive IAP measurement techniques are summarized in Table 1 and Fig. 27.

**Table 1:** Potential non-invasive intra-abdominal pressure (IAP) measurement techniques

Measurement technique	Concept behind the measurement technique	Advantages	Disadvantages	Compatibility with the specific ICU environment
Strain gauge	Correlation between IAP and external torque around mid-lumbar level	Simple Low-cost	High motion sensitivity Low signal to noise ratio	Restricted applications due to patient positioning during IAP measurement
Respiratory Inductance Plethysmography	Correlation between IAP and IAV	Simple Low-cost	Lack of reliability Motion sensitivity	Compatible
Tensiometry	Correlation between AWT and IAP	Fast Inexpensive High accuracy	Lack of standardized tensiometer	Compatible
Ultrasound tonometry	Correlation between IAP and applied force on the ultrasound probe	Relatively inexpensive Fast Easily reproducible	Need of coupling gel Low accuracy	Compatible
Ultrasound assessment in combination with external pressure	Correlation between IAP and external force on abdominal wall	Fast Inexpensive Reproducible	The need of coupling gel Measurement limitations	Compatible
Doppler ultrasound	Correlation between IAP and blood flow	Relatively inexpensive Fast	Lack of accuracy	Compatible
Laser ultrasound	Correlation between IAP and wavelength of the reflected/transmitted pulse	No need for coupling gel, vast dynamic range for measurement	Relatively expensive, lack of proof of concept	Contactless and ideal for use in ICU
Bioimpedance	Correlation between IAP and electrical impedance of abdominal wall	High accuracy	Lack of sensitivity for IAP values higher than 7 mmHg	Restricted applications due to the impedance electrode placement
Microwave reflectometry	Correlation between IAP and reflection response (S11) of microwaves	High accuracy Contactless	The need for further validations	Contactless and ideal for use in ICU
Digital Image Correlation	Optical tracking and image registration of abdominal compartment	High accuracy	Expensive Complex set-up Lack of proof of concept	Restricted applications due to its equipment placement in ICU
Wireless motility capsule	Direct measurement of intragastric pressure	–	Expensive Low accuracy	Compatible

The second column manifests the logic behind each measurement technique, while the advantages and disadvantages of each method are shown in the third and fourth column, respectively. The last column is also related to the possibility of using each measurement method in the specific conditions of ICU (compatibility with other medical equipment, monitoring systems, etc.). Intra-abdominal volume, abdominal wall, and abdominal wall tension are abbreviated as IAV, AW, and AWT, respectively

*AWT* abdominal wall tension, *IAP* intra-abdominal pressure, *IAV* intra-abdominal volume, *ICU* intensive care unit

Currently, wireless motility capsules, digital image correlation, and laser ultrasound cannot be proposed as the best measurement techniques, mainly due to the lack of validation and clinical research. Respiratory inductance plethysmography, the use of a strain gauge and Doppler ultrasound tonometry for IAP estimation are not reliable or accurate enough to be used in clinical practice. As mentioned before, it is better to combine these techniques with more accurate ones. For instance, it might be useful to use these techniques for the primary detection of IAH, in combination with a standard transvesical measurement technique for confirmation, and more accurate follow-up measurements. Tensiometry showed acceptable results, however, since there is no standardized tensiometer for IAP measurement, it seems more pragmatic to focus on the remaining techniques. For IAP measurement up to 7 mmHg, bioimpedance is one of the best methods that can be used. But, as mentioned before, this technique is not able to monitor clinically important IAP changes (pressures > 12 mmHg). Finally, ultrasound assessment in combination with external pressure and microwave reflectometry seem the most promising non-invasive IAP measurement methods that showed a good correlation with IAP as well as a relatively higher accuracy in the range of the pressure values that are expected to occur in patients hospitalized in the ICU. However, by ultrasound assessment, we can only monitor the IAP that is equal to the external pressure exerted on the abdominal wall, since the peritoneal rebound occurs only when the IAP is equal to or above the external pressure. Furthermore, the external pressure exerted on the abdominal wall needs to be standardized in the future. This is another downside of this measurement method that restricts its broad application at the bedside.

Also, it should be pointed out that all these reviewed techniques are able to monitor the IAP changes rather than estimating the absolute IAP value. Thus, we need to know the initial IAP value in order to monitor the IAP trend. In our perspective, estimation of the initial IAP can be done based on (for instance) the initial thickness of the different layers of the abdominal wall in combination with a gold standard reference method (e.g. bladder pressure). However, more investigation needs to be done in the future looking for correlations between specific body parameters and IAP in order to predict the baseline IAP value with high accuracy and reliability.

## Data Availability

All data generated or analyzed during this study are included in this review paper (and its supplementary information files).
